# Biophysical Tools and Concepts Enable Understanding of Asexual Blood Stage Malaria

**DOI:** 10.3389/fcimb.2022.908241

**Published:** 2022-05-31

**Authors:** Viola Introini, Matt A. Govendir, Julian C. Rayner, Pietro Cicuta, Maria Bernabeu

**Affiliations:** ^1^Cambridge Institute for Medical Research, University of Cambridge, Cambridge, United Kingdom; ^2^Cavendish Laboratory, University of Cambridge, Cambridge, United Kingdom; ^3^European Molecular Biology Laboratory (EMBL) Barcelona, Barcelona, Spain

**Keywords:** malaria, biophysics, imaging, *Plasmodium*, cytoadhesion, microfluidics, mechanobiology

## Abstract

Forces and mechanical properties of cells and tissues set constraints on biological functions, and are key determinants of human physiology. Changes in cell mechanics may arise from disease, or directly contribute to pathogenesis. Malaria gives many striking examples. *Plasmodium* parasites, the causative agents of malaria, are single-celled organisms that cannot survive outside their hosts; thus, thost-pathogen interactions are fundamental for parasite’s biological success and to the host response to infection. These interactions are often combinations of biochemical and mechanical factors, but most research focuses on the molecular side. However, *Plasmodium* infection of human red blood cells leads to changes in their mechanical properties, which has a crucial impact on disease pathogenesis because of the interaction of infected red blood cells with other human tissues through various adhesion mechanisms, which can be probed and modelled with biophysical techniques. Recently, natural polymorphisms affecting red blood cell biomechanics have also been shown to protect human populations, highlighting the potential of understanding biomechanical factors to inform future vaccines and drug development. Here we review biophysical techniques that have revealed new aspects of *Plasmodium falciparum* invasion of red blood cells and cytoadhesion of infected cells to the host vasculature. These mechanisms occur differently across *Plasmodium* species and are linked to malaria pathogenesis. We highlight promising techniques from the fields of bioengineering, immunomechanics, and soft matter physics that could be beneficial for studying malaria. Some approaches might also be applied to other phases of the malaria lifecycle and to apicomplexan infections with complex host-pathogen interactions.

## Introduction

**Figure 1:**
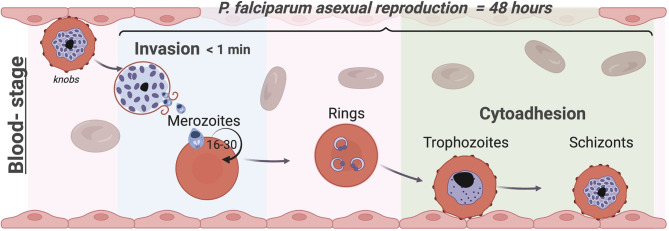
The blood stage of *Plasmodium falciparum*. Life cycles of Apicomplexa involve asexual and sexual reproduction stages in different hosts. *Plasmodium*’s asexual replication in humans occurs in the liver and bloodstream while the sexual stages occur in the mosquito. Parasites are injected into the human skin through the bite of an infected female *Anopheles* mosquito (Frevert et al., 2005; Amino et al., 2006); they first travel to the liver (Menard et al., 2013), where they proliferate into merozoites, before being released into the blood stream. Merozoites invade circulating RBCs initiating the blood stage (Cowman et al., 2017). While invasion typically occurs in less than a minute, within the RBC the parasite undergoes maturation in approximately 48 hours from a ring-stage to a trophozoite by digesting haemoglobin before starting nuclear divisions into daughter merozoites as schizont. At this point, the iRBCs rupture releasing up to 30 daughter merozoites (Rudlaff et al., 2020) producing an exponential growth of parasites inside the host that causes all the symptoms of the disease. To avoid clearance by the spleen, trophozoites and schizonts of *P. falciparum* adhere to the endothelium and to other uninfected RBCs (rosettes) and sequester in the vasculature, leading to severe complications associated with this malaria parasite such as cerebral and placental malaria. In each cycle, 5-10% of parasites develop into the sexual forms and are transmitted to the mosquito vector to complete the life cycle (Frischknecht and Matuschewski, 2017). Image made using ^©^BioRender (https://biorender.com).

Malaria is an infectious disease caused by multiple species of *Plasmodium* protozoan parasites of the phylum Apicomplexa. Malaria killed more than half a million people in 2020 (World Malaria Report, 2021), with *Plasmodium falciparum* causing the overwhelming majority of deaths. Although *Plasmodium* research has typically focussed on the genetic and biochemical determinants of disease, recent advances in imaging and mechanobiology offer the opportunity to explore how mechanics and the environment contribute to various aspects of the *Plasmodium* life cycle and pathogenesis, in particular the blood stage reported in **Figure 1**. Multiple biophysical tools are now being applied to understanding parasite-host interactions in malaria, particularly the mechanisms of highly dynamic processes such as parasite-red blood cell (RBC) invasion, and cytoadhesion, where the infected red blood cell (iRBC) interacts with the microvasculature and other human cells; both invasion and cytoadhesion play a critical role in severe malaria pathogenesis.

Progress in live imaging has been at the heart of recent discoveries: improvements on real-time and time-lapse microscopy speed and resolution have been crucial in imaging *Plasmodium* parasites in *in vitro* and *in vivo* experimental settings. Likewise, many biophysical methods have been applied to quantitatively characterise the mechanical properties and the adhesion force of healthy RBCs and *Plasmodium*-infected RBCs in bulk and at single-cell level [refer to (Depond et al., 2019)]: all terms and the relative technologies presented in this work are summarised in the Glossary **Table 1**. Techniques to probe and measure forces at the cell scale include micropipette aspiration (Mohandas et al., 1984), atomic force microscopy (Sinha et al., 2015), and optical tweezers (Crick et al., 2014). Recently, membrane flickering spectroscopy has been used as a powerful tool to elucidate the role of RBC mechanics in disease protection (Kariuki et al., 2020). To address the intricacies of parasite and human biology, sophisticated microfluidic devices have been engineered to tune physical and biological parameters in well-controlled reproducible assays while maintaining aspects of physiological relevance, and create *in vitro* models that more accurately reproduce *in vivo* tissue microenvironments.

**Table 1:** Red blood cell biophysical properties, techniques, and effects on malaria.

RBC properties (Unit)	Definition	Techniques (References); Values	Influence on invasion	Influence on intracellular growth and cytoadherence
CELL DEFORMABILITY/RIGIDITY/STIFFNESS (deformability or elongation index, dimensionless)	General term that describes the ability of a soft body to change its shape in response to an external force In RBCs it depends on: (i) surface area-to-volume ratio (ii) intracellular viscosity (haemoglobin) (iii) membrane viscoelasticity Depending on the experimental protocol, it correlates to a combination of RBC shape and size, cell viscosity, membrane bending and tension	Ektacytometry (Bessis et al., 1980; Mohandas et al., 1984; Chasis et al., 1985; Schrier et al., 1989; Deplaine et al., 2011; Sisquella et al., 2017; Barber et al., 2018; Ebel et al., 2021) (osmotic gradient ektacytometry); deformability index 0-0.65 (shear stress from 0.3 to 30 Pa) Microsphiltration (Deplaine et al., 2011)and filtration (Reid et al., 1976); retention or enrichment rates (from < 10 to 100%) Microfluidics (Glodek et al., 2010) time of passage, shape deformation and recovery through restrictions	Decrease in deformability reduces invasion in Malayan ovalocytes (Mohandas et al., 1984) Deformability reduced in RBCs pretreated with glycophorin A-specific antibodies (Chasis et al., 1985), but on the contrary binding of *Pf* ligand EBA-175 RII recombinant protein to RBCs increases deformability (Sisquella et al., 2017) CR1 ligation increases RBC membrane deformability (Glodek et al., 2010)	Reduced elongation index in RBCs from *P. falciparum (Pf)* and *P. knowlesi (Pk)*-infected patients (Barber et al., 2018)
SURFACE AREA (μm^2^)	Membrane surface area is maintained by cohesion between the lipid bilayer and the spectrin-based membrane skeleton	Image flow cytometry (Safeukui et al., 2013) 4D Lattice light-sheet microscopy (Geoghegan et al., 2021); before invasion 135-150 μm^2^, after invasion 115-135 μm^2^ Microfluidics (Herricks et al., 2009); 140-160 μm^2^ Laminar shear flow (Suwanarusk et al., 2004); 50 μm^2^ for *P. vivax* rings – 90 μm^2^ for *P. vivax* schizonts Micropipette aspiration (Nash et al., 1989);130-150 μm^2^ Confocal fluorescence microscopy and 3D reconstruction (Waldecker et al., 2017); normalised surface area	Stepwise reduction in RBC surface area is related to the total sum of surface area due to the PVM of each parasite (Geoghegan et al., 2021)	*Pf*-iRBCs have a smaller surface area: 9.6% (rings) and 14.2% (trophozoites) surface area loss (Safeukui et al., 2013; Waldecker et al., 2017) Measure *Pf*-iRBC surface area over 48-hour time course (Herricks et al., 2009) Decreased surface area to volume ratio (Nash et al., 1989) Increase surface area for *P. vivax (Pv)*-iRBCs *(* Suwanarusk et al., 2004)
VOLUME (μm^3^, fL)	The volume is determined by the water content and its homeostasis is regulated by various membrane-associated ion transporters and channels	4D Lattice light-sheet microscopy (Geoghegan et al., 2021); reduced volume increases after merozoite penetration Microfluidics (Herricks et al., 2009); 60-120 μm^3^ for uninfected RBCs - 80-150 μm^3^ for iRBCs Micropipette aspiration (Nash et al., 1989); 90 fL for uninfected RBCs -100 fL for iRBCs Confocal fluorescence microscopy and 3D reconstruction (Esposito et al., 2010; Waldecker et al., 2017); reduced volume 0.5 to 0.9 after 48 hours	Echinocytosis starts immediately after parasite penetration (Geoghegan et al., 2021)	Volume of *Pf*-iRBCs remains almost constant during parasite maturation (Esposito et al., 2010), but Nash 1989 (Nash et al., 1989) found 11.3% increase volume in rings Measure *Pf*-iRBC volume over 48-hour time course (Herricks et al., 2009; Waldecker et al., 2017)
OSMOTIC STRESS (Pa) (Osmotic fragility: the propensity of a red cell to lyse while absorbing water under hypotonic stress - reflects biophysical membrane properties)	Pressure due to imbalance of solutes across a semipermeable membrane A sudden change in the solute concentration around a cell will cause a rapid movement of water across its cell membrane through osmosis: change in cell size, shape, hydration, viscosity, haemoglobin concentration, crowding May correlate to a combination of RBC shape and size, cell viscosity, membrane bending and tension	Osmotic fragility tests (Ebel et al., 2021); % haemolysis/haemoglobin concentration (Tiffert et al., 2005)	Dehydrated RBCs become denser and less susceptible to *Pf* invasion (Tiffert et al., 2005)	Higher red cell osmotic fragility sustains slower *Pf* growth *in vitro (* Ebel et al., 2021)
VISCOSITY (*η*, Pa s) (Dissipation in dynamical response. Note that in principle each of elastic shear modulus, Young’s modulus, and bending modulus has a dynamical dissipative counterpart, but they have not been measured individually in RBCs)	Resistance to deformation at a given rate Viscosity = plasma membrane viscosity + cytoplasm viscosity (mainly haemoglobin) May correlate to a combination of RBC shape and size, membrane bending and tension	Flickering spectroscopy (Evans et al., 2008; Betz et al., 2009; Yoon et al., 2009; Froühlich et al., 2019; Kariuki et al., 2020; Davies et al., 2020); 0.01-10 Pa s Micropipette aspiration (Evans et al., 1984) AFM (Dulinska et al., 2006) High-frequency electric field (Engelhardt et al., 1984)	Higher viscosity in dehydrated RBCs correlates to reduced *Pf* invasion (Tiffert et al., 2005)	Viscosity increases during *Pf* intraerythrocytic development, no difference in the kinase FIKK 4.1 knock-out lines (Froühlich et al., 2019; Davies et al., 2020)
MEMBRANE SHEAR MODULUS (μ, N/m^2^, mDynes/cm^2^ or N/m and mDynes/cm for 2D)	A material property that describes the ability of a soft body to change its shape in response to an external force It is well defined for a homogeneous material (3D block or 2D sheet) but in the context of a red blood cell it can correlate to a combination of RBC shape and size, membrane bending and tension	Optical tweezers (Suresh et al., 2005; Mills et al., 2007); from 5 μN/m (uninfected RBCs) to 53 μN/m (schizonts) Micropipette aspiration (Nash et al., 1989; Glenister et al., 2002; Rug et al., 2006); 5-20 mDynes/cm^2^ - up to 50 pN/μm for *Pk*-iRBCs *(* Barber et al., 2018) Laminar shear flow (Suwanarusk et al., 2004); 3-5 mDynes/cm^2^ Diffraction phase microscopy (Park et al., 2008); from 6 μN/m (uninfected RBCs) to 100 μN/m (schizonts) Holographic microscopy (Byeon et al., 2015); 5-25 μN/m from healthy to trophozoites High-frequency electric field (Engelhardt et al., 1984); 7x10^-6^ N/m AFM (Sinha et al., 2015); RBCs treated with different compounds 5-15 μN/m	Decrease in deformability reduces invasion in Malayan ovalocytes (Mohandas et al., 1984) RBCs with stiffened membranes do not support merozoite penetration (Sinha et al., 2015)	Shear modulus increases during *Pf* parasite development (Suresh et al., 2005; Park et al., 2008; Byeon et al., 2015) -supported by simulations (Hosseini and Feng, 2012; Zhang et al., 2015) and change with temperature (Mills et al., 2007; Park et al., 2008) Knobs are responsible for increasing *Pf-*iRBC rigidity (Glenister et al., 2002) Decrease in *Pv-*iRBCs *(* Suwanarusk et al., 2004), increase in both human and macaque *Pk-*iRBCs *(* Barber et al., 2018)
YOUNG’S MODULUS (E, N/m^2^ or N/m for 2D)	Given by stress/strain in a specific deformation geometry - Measures the resistance of a material to elastic deformations It is proportional to the elastic shear modulus for a given geometry of deformation	AFM (Dulinska et al., 2006; Sisquella et al., 2017; Aniweh et al., 2017); 19-33 kPa	Binding of *Pf* ligand EBA-175 RII recombinant protein shows a dramatic reduction of RBC Young’s modulus, a decrease is also seen for Rh4-CR1 and Rh5-Basigin binding (Sisquella et al., 2017; Aniweh et al., 2017)	Increase during *Pf-*iRBC development (Ademiloye et al., 2017)
BENDING MODULUS (or bending rigidity, ĸ, J)	Energy required to bend a membrane, by changing its curvature Depends on membrane asymmetry, thickness (including cytoskeleton and membrane proteins), lipid composition, lipid packing and order	Flickering spectroscopy (Popescu et al., 2006; Evans et al., 2008; Betz et al., 2009; Yoon et al., 2009; Koch et al., 2017; Froühlich et al., 2019; Kariuki et al., 2020; Davies et al., 2020) 10^-20^ - 9x10^-19^ J Micropipette aspiration (Evans et al., 1984);	Decrease in membrane bending increases invasion efficiency (Koch et al., 2017)	No significant change in *Pf* rings, higher for trophozoites (Froühlich et al., 2019; Davies et al., 2020)
TENSION (or membrane extensional rigidity, σ, N/m)	Force needed to stretch the membrane Different regimes depending on the ‘excess surface area’ of the cell membrane and on membrane-cytoskeleton adhesion (Lim et al., 2002). Once the membrane is taught, then depends on lipid composition	Flickering analysis (Popescu et al., 2006; Evans et al., 2008; Betz et al., 2009; Yoon et al., 2009; Koch et al., 2017; Froühlich et al., 2019; Kariuki et al., 2020; Davies et al., 2020); 10^-8^ - 10^-5^ N/m Optical tweezers (Yoon et al., 2008); stiffness = 10-30 pN/μm depending on the strain rate	Correlation between increase tension and reduce *Pf* invasion (Kariuki et al., 2020) (Modelling Hillringhaus et al., 2009)	Increase during *Pf* development (Froühlich et al., 2019; Davies et al., 2020) Slightly reduced tension in knobless trophozoites (Froühlich et al., 2019)
ELECTRIC SURFACE CHARGE (or membrane potential mV)	Sialylated glycoproteins of the RBC membrane are responsible for a negatively charged surface	Surface potential microscope (Aikawa, 1997; Akaki et al., 2002); charge of *Pf* knobs: +20 mV, free merozoite apex: +65.10 mV Optical tweezer (Fontes et al., 2008); zeta potential RBC: -12.5 mV Transmembrane distribution of radiolabelled anions (Mikkelsen et al., 1982); uninfected RBCs: -10 mV, rings: -16 mV, schizonts: -35 mV (Mikkelsen et al., 1986). *Pf* schizonts free of host cell membrane: -90 mV	Merozoite apical end is positively charged while the body is negatively charged (Aikawa, 1997) - the apex binds to the negatively charged RBCs during invasion	Knobs of *Pf*-iRBCs are positively charged, thus, facilitating adhesion to the endothelial cells which have a negative surface charge (Hillringhaus et al., 2009) Membrane potential is -16 mV for rings and -35 mV for late stage iRBCs (Fontes et al., 2008)

These concepts fit within the broader efforts in “mechanobiology”, i.e. the effort to generally understand the relationship between a cell and its surroundings. Here we use this term to specifically refer to how *Plasmodium* parasites respond to or cause changes in host cell mechanics, physical forces, and substrate structure, and if mechanical pathways influence the infected cells or host tissues.

In this review, we focus on disruptive technologies that have been used to investigate the physical traits of *P. falciparum*-human interactions during invasion (**Section 1**) and cytoadhesion (**Section 2**), where such technologies have generated paradigm shifts in our knowledge of malaria and important insights into potential new intervention strategies. **Section 3** will describe the biomechanical determinants of invasion and cytoadhesion in other species of *Plasmodium*. This review takes a problem-oriented approach, to show how specific research questions in malaria have been addressed using biophysical techniques, hoping to inform the reader in the importance of biophysics in infection, and inspire to adopt similar approaches to tackle their own research questions.

## Section 1: Mechanics of Red Blood Cell Invasion

Malaria clinical symptoms arise during the *Plasmodium* blood stage, which starts when merozoites recognise and invade human RBCs. The invasion process is essential for parasite survival and replication, and consists of a sequence of mechanical and biochemical events that are precisely timed and tightly regulated. During invasion parasites are extracellular and hence exposed to the antibody mediated immune system, representing a potential target for vaccine and drug development. Advanced microscopy techniques such as cryo-electron tomography (Hanssen et al., 2013), cryo-X-ray tomography (Hanssen et al., 2011), electron microscopy (Kumar et al., 2017), and super-resolution structured illumination microscopy (Riglar et al., 2011) have been crucial to unveil the structure of merozoite organelles involved in invasion, and the molecular mechanisms that determine the irreversible attachment between merozoites and RBCs (Mauritz et al., 2010; Cho et al., 2012). However, these techniques operate with fixed samples providing static snapshots, which is not ideal for a highly dynamic and rapid process such as invasion, which is usually complete in less than a minute. Therefore, a main challenge in the field has been to follow the egress-invasion sequence in real time, and this is typically achieved with live imaging in brightfield, phase contrast, differential interference contrast (DIC), and epifluorescence (Glushakova et al., 2005; Gilson and Crabb, 2009; Crick et al., 2013; Introini et al., 2018a).

### Subsection 1.1. Live Microscopy Reveals the Egress-Invasion Sequence and Intraerythrocytic Cycle

**Figure 2:**
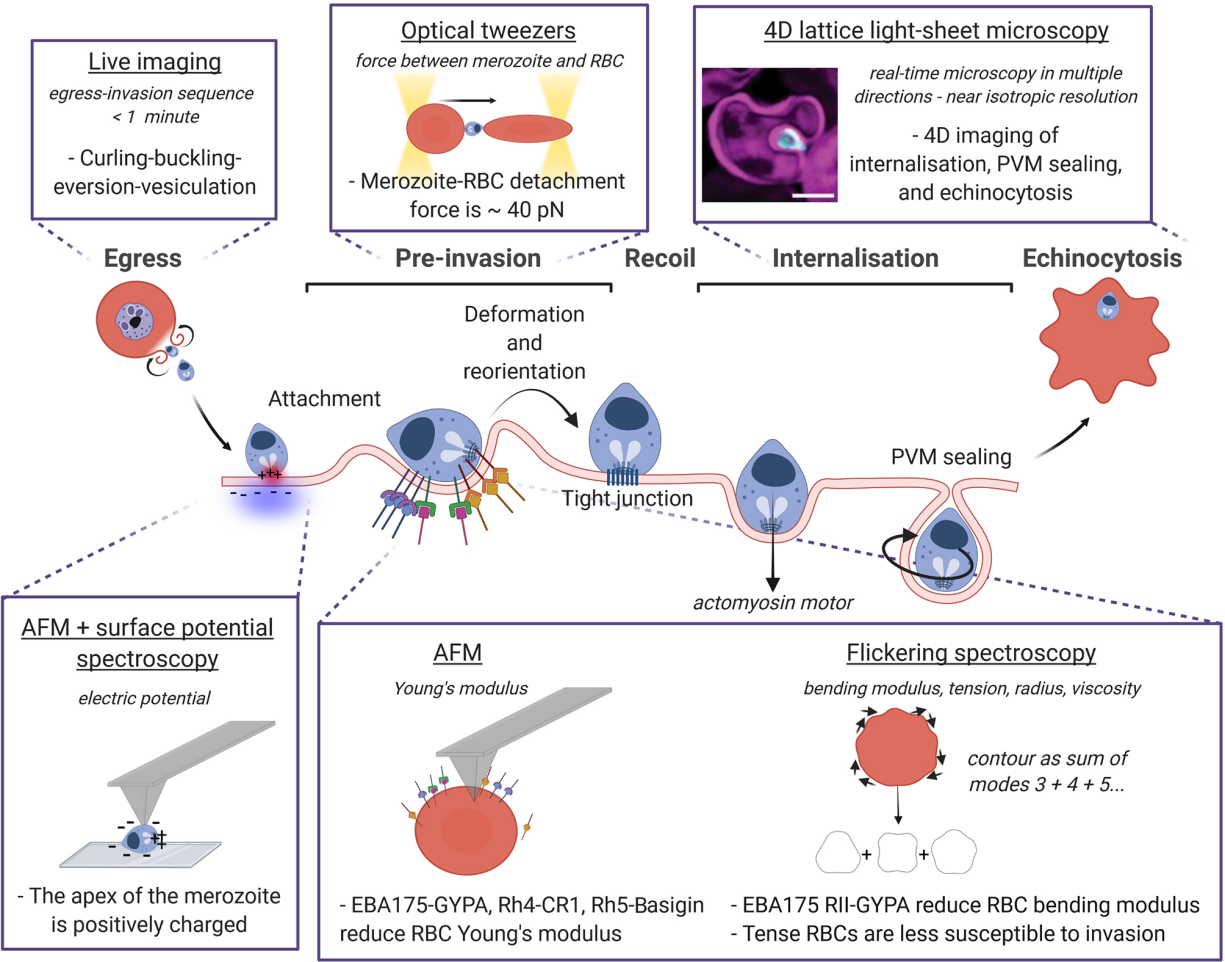
Biophysical techniques and methods to probe the mechanics of *P. falciparum* invasion. The sequence egress, pre-invasion, recoil phase, internalisation and finally echinocytosis is represented with a focus on the biophysical approaches used to measure the mechanical properties of the RBC and the parasite, and their interactions. The technique is underlined, the quantity measured with the relative technique is in italics, and the dashed sentences report the main findings. Image made using ^©^BioRender (https://biorender.com). LLSM image was taken from Geoghegan et al. (Geoghegan et al., 2021).

Over the last two decades, progress on the video temporal and spatial resolution permitted the recording of the kinetics of merozoite egress and subsequent invasion of RBCs up to hundreds of frames per second with sub-μm resolution, and has been used to assess the effect of antibodies, enzyme inhibitors, and ion signalling on the invasion process (Glushakova et al., 2005; Gilson and Crabb, 2009; Crick et al., 2013; Weiss et al., 2015; Bustamante et al., 2017). The egress was initially described in the literature as an “explosive” phenomenon (Lew, 2005; Mauritz et al., 2010), but Abkarian et al. (Abkarian et al., 2011) revealed a detailed dynamic morphology of the host cell membrane rupture using high-speed video microscopy, as represented in **Figure 2**. At the instant of schizont rupture, a pore opening in the red cell membrane allows only 1 or 2 merozoites to emerge. Once the pore reaches a critical radius, the host cell membrane rapidly curls outwards to form a circular toroid around the initial opening, and then buckles, undergoing eversion to push out and disperse the rest of the merozoites. Finally, the everted membrane spontaneously forms vesicles (Lew, 2011). This curling-buckling-eversion-vesiculation sequence is completed in about 400-800 ms (Callan-Jones et al., 2012; Braun-Breton and Abkarian, 2016) and resembles, on a shorter time scale, the spontaneous vesiculation of uninfected RBCs found previously by Lew et al. (Lew et al., 1988; Tiffert and Lew, 2014). This similarity may be evidence of the parasite exploiting an intrinsic host cell property and remodelling the cortical cytoskeleton for a rapid response during egress (Kabaso et al., 2010).

The three main phases in which invasion is usually divided i) pre-invasion, ii) recoil phase, and iii) internalisation (Gilson and Crabb, 2009; Groomes et al., 2022) are described in **Figure 2**, highlighting the biophysical techniques developed to measure both the parasite and the RBC physical quantities at each phase. During pre-invasion, which lasts between 2-50 s, merozoites make reversible contacts with RBCs triggering transient deformations of the cell membrane from the contact site. The merozoite re-positions itself on the surface of the RBC until it is apically aligned, and binds to the RBC using adhesins on the merozoite coat (Tham et al., 2015; Weiss et al., 2016). After a recoil phase where RBC deformations cease and the biconcave shape is restored, the secretory organelles located in the merozoite apex release ligands into the RBC and the merozoite is irreversibly attached (Treeck et al., 2009; Crosnier et al., 2011). The tight junction is then formed, and penetration begins driven by the merozoite actomyosin motor. Merozoite internalisation is frequently, but not always, accompanied by echinocytosis (taking approximately 5-15 minutes (Gilson and Crabb, 2009) where the RBC outer surface curls and forms spikes, before restoring their original biconcave shape with the parasite-ring inside. Transition of RBCs from discocytes to echinocytes results from the expansion of the outer leaflet of the plasma membrane with respect to the inner one (Lim et al., 2002), producing convex structures on the cell surface (echinocytic spicules) to accommodate the extra area, and resulting in increased bending modulus, shear modulus, and surface area (Park et al., 2010) (**Table 1**). Computational modelling of this invasion processes is a huge and still open challenge since invasion ligand expression is controlled in time and space, and ligand-receptor interaction binding rates are modulated by membrane properties (Mognetti et al., 2019; Hillringhaus et al., 2020).

The latest imaging upgrade to investigate *Plasmodium* invasion is given by the 4D lattice light-sheet microscopy (LLSM), described by the pioneering work of Chen and colleagues (Chen et al., 2014). LLSM provides volumetric information at hundreds of images per second, and a distinct advantage of this method over the conventional 2D microscopy consists of looking at parasites and RBCs from multiple orientations given by the near isotropic resolution (Geoghegan et al., 2021). Compared to confocal laser scanning microscopy, the thinness of the sheet produces a high axial resolution (the ability to discern between two points at different depth along the optical axis of the imaging objective) and larger field of view, a better signal-to-noise ratio when using multiple fluorescent probes, and less photobleaching. LLSM has succeeded in characterising all stages of invasion quantitatively, including parasite internalisation and parasitophorous vacuole membrane (PVM) formation (Geoghegan et al., 2021) (**Figure 2**). Despite many shape changes throughout the invasion process, the membrane area and volume of host RBCs were previously thought to remain essentially unchanged (Mauritz et al., 2009; Waldecker et al., 2017). Here the authors showed a stepwise decrease in RBC surface area which coincides with the total sum of surface areas lost for the PVM of each parasite invading the same RBC.

Recent imaging of CRISPR Cas9 engineered parasite lines with fluorescent tags (Lee et al., 2019), has boosted the study of specific ligand-receptor interactions at the molecular level. This has permitted the localisation of apical ligands that take part in the invasion (Treeck et al., 2009), and showed that the invasion proceeds with the wider end of the merozoite leading, rather than the narrower pointed end, highlighting a remarkable motion of the merozoite that until now was mostly overlooked (Yahata et al., 2021). Moreover, machine learning algorithms for image recognition have been employed to automatically detect infected cells and their stage (Davidson et al., 2021).

One challenge for live-cell imaging is the long, repeated exposure to light which affects both the fragile parasites and the RBCs. Phototoxicity induces lysis of the acidic food vacuole of the parasites (Wissing et al., 2002) and damages RBC haemoglobin inducing autofluorescence (Shrirao et al., 2021), making it very difficult to follow the parasites during the entire intraerythrocytic cycle. Using DIC and confocal microscopy, Gruring et al. (Grüring et al., 2011) were able to visualise the 48-hour development of *P. falciparum*, elucidating the trafficking mechanism of exported proteins to the host membrane and revealing a fascinating morphology of the parasite at the ring stage. Contrary to the familiar features visible with Giemsa staining, rings are not always circular but assume transient amoeboid forms during the first 5-10 hours post invasion. This phenomenon would require further investigations in real time to explain this initial amoeboid cytoplasm.

### Subsection 1.2. Biophysical Analysis of Receptor-Ligand Interactions

The initial merozoite-red cell adherence can occur at any point on the merozoite surface due to low-affinity and reversible interactions between Merozoite Surface Proteins (MSPs) and receptors on the red cell membrane - with coat filaments of 20-150 nm length joining the two surfaces (Bannister et al., 1986). Subsequent reorientation and final attachment require Erythrocyte-Binding-Like (EBL), Reticulocyte binding homologous (Rh), and AMA-1 ligands that span the red cell membrane and link to the merozoite cytoskeleton. These adhesive ligands are secreted from the organelles located at the apex of the 1 μm ovoidal merozoite, which was for a long time presented and modelled as being the narrow end, but recently this depiction has been questioned (Yahata et al., 2021). In hindsight, if the parasite is pear-shaped it will rotate to have the less curved side attached to the membrane. This will reduce bending energy, and allow a larger adhesive area per merozoite. The merozoite shape and orientation therefore play a key role in the mechanics of invasion, which needs further investigation.

Beyond mediating attachment, such ligand families can also transduce signals and modify the physical properties of the host cell membrane (described in **Table 1**). For example, recombinant proteins Rh5 binding to Basigin mediate Ca^2+^ signal in the RBC triggering phosphorylation of specific proteins and the subsequent rearrangements of the RBC cytoskeleton that facilitate the insertion and anchoring of rhoptry proteins and junction formation (Aniweh et al., 2017), and increases deformability, i.e. decreases the Young’s modulus of RBCs, albeit to a lesser extent than binding of MSPs, Rh4, or EBA175, hence favouring invasion (Sisquella et al., 2017) (**Figure 2**). EBA175 RII binding to the RBC receptor Glycophorin A (GYPA) induces a reduction in the host cell bending modulus that increases invasion efficiency, by lowering the energy necessary to bend the RBC surface and facilitate merozoite attachment. The possible caveat is that the local concentration of EBA175 (or any other ligand) on the merozoite surface is unknown, so it is hard to predict how well the experimental set up mimics the actual host RBC-merozoite interaction during invasion. These measurements were performed with AFM, and confirmed using flickering spectroscopy (Koch et al., 2017), as described in **Figure 2**. Flickering spectroscopy is an optical technique that extracts the cell bending modulus (the energy required to bend the membrane) and tension (the resistance of the membrane to stretch) by analysing the plasma membrane thermal fluctuations. By extending this approach into the dynamical domain and measuring the relaxation rates, it is possible to extract the cytosolic viscosity (Helfrich and Servuss, 1984; Monzel and Sengupta, 2016) (example in **Figure 2**) Flickering experiments served to investigate the biophysical properties of RBCs depending on oxygenation level, cell morphology, hydration state (Yoon et al., 2009), glucose concentration (Tapia et al., 2021) and parasite infection (Davies et al., 2020); as well as exploring the biophysical impact of malaria-protective polymorphisms affecting RBCs (such as hemoglobinopathies S and C (Froühlich et al., 2019), Dantu blood group (Kariuki et al., 2020) and beta-thalassaemia Introini et al., 2022). The advantages of this method to measure deformability over the more traditional tools, as AFM and optical tweezers, are i) the easy set-up where only a good quality camera is required, ii) the non-invasiveness since samples are not directly manoeuvred, and finally iii) the ability to decouple bending modulus and tension, while other techniques yield the effective shear modulus of the composite system (Kariuki et al., 2020) (see **Table 1**). It is notable that the theoretical assumptions behind this method are valid only for circular-like closed objects like vesicles and RBCs.

But how strong is the attachment between the RBC and the invading merozoite? Crick *et al.* measured for the first time the detachment force between a merozoite and an RBC using optical tweezers (40 ± 8 pN) (Crick et al., 2014). Within few seconds from egress, a merozoite is brought in contact with nearby RBCs forming a chain of RBC-merozoite-RBC, and the two trapped RBCs are pulled away simultaneously using optical tweezers (sketch in **Figure 2**). For a given rate of extension, RBC have been shown to behave like linear springs, i.e. they have a well -stiffness. Thus, the detachment force can be calculated knowing this RBC stiffness and observing the maximum elongation of the cells just before detachment. Generally used for mechanical measurements, optical tweezers are a powerful method for positioning μm-size colloids and cells, and therefore optimal for manipulating individual merozoites and RBCs during invasion (Mauritz et al., 2010). The main difficulty of this technique comes from the low yield due to the short viability of free *P. falciparu*m merozoites after egress (< 2 minutes). Only treatments like heparin that block invasion at the early stage show a reduction in the detachment force attesting that the force measured comes from ligands and receptors participating in the pre-invasion phase (Crick et al., 2014). The electrostatic attraction of the positively charged apex of the merozoite towards the negative surface of the RBC measured using AFM with surface potential microscopy (Akaki et al., 2002) adds to the force generated by EBL and Rh ligands, although the exact ligand responsible for the measured force has not been identified yet, as most EBL and Rh ligands mediating attachment have a redundant function (Menard et al., 2013; Weiss et al., 2015) (**Figure 2**).

A hallmark of invasion videos is the dynamic deformations of the RBC membrane elicited by merozoite adhesion during pre-invasion. These RBC deformations wrap the merozoites with different strength which correlate with successful invasion (Weiss et al., 2015; Kariuki et al., 2020; Geoghegan et al., 2021) and have been categorised visually from weak to strong by using a scale from 0 to 3 (Weiss et al., 2015). Calcium influx from the merozoite to the RBC was hypothesised to trigger deformations to assist parasite reorientation (Lew and Tiffert, 2007), but this has been disproved by using simultaneously bright-field and fluorescence live microscopy, suggesting instead that local membrane deformations might simply be a passive byproduct of parasite adhesion to the RBC (Introini et al., 2018a; Hillringhaus et al., 2020). This view was supported by Dasgupta et al.’s numerical simulations (Dasgupta et al., 2014) showing that merozoite shape and apical adhesion gradient contributed extensively to wrapping, with an indentation force during the early stage of the invagination estimated of 13 pN (Abdalrahman and Franz, 2017). This is in fact what happens when placing spent merozoites close to RBCs using optical tweezers, they in fact had lost the ability to invade 2-3 minutes post-egress but nonetheless adhere to RBCs and still induce transient local membrane deformations (Crick et al., 2013; Crick et al., 2014).

Altogether, these approaches show that biophysical measurements are necessary to understand how the strength of interaction between specific ligands and RBC receptors affects the host cell mechanics.

### Section 1.3. Contribution of Host Cell Mechanics to Invasion

Not only do merozoite interactions modify the biomechanical properties of the RBC to facilitate parasite entry, but naturally occurring mutations in proteins of RBC membrane can impact the host cell biophysics and confer protection against malaria. Malaria has provided a strong selective force on the human genome, leading to the selection of many polymorphisms that provide some level of protection against severe disease. Given that all the symptoms and pathology of malaria occur during the intraerythrocytic stage, unsurprisingly most of these polymorphisms relate to the structure and function of red blood cells (Kariuki and Williams, 2020). *Plasmodium* merozoites are less able to invade RBCs with higher membrane rigidity (Mohandas et al., 1984; Chasis et al., 1985), as documented in a plethora of mutations in the RBC membrane associated with protection against malaria (Mohandas et al., 1984; Froühlich et al., 2019). A recent example is the rare Dantu variant, present at appreciable frequency almost only in East Kenya, which is caused by a rearrangement of Glycophorin A and B genes that results in the expression of a hybrid Dantu protein with a GYPA extracellular domain/GYPB tail fusion that does not interact with the RBC cytoskeleton (Malaria Genomic Epidemiology Network., 2015; Leffler et al., 2017; Ndila et al., 2018). By combining real-time imaging with flickering spectroscopy, Kariuki *et al. (* Kariuki et al., 2020) were able to quantitate for the first time both the biophysical properties of the host cell and the invasion outcome by *P. falciparum* merozoites, and compare these properties across Dantu genotypes. These data suggested that there is a threshold for RBC membrane tension, above which the chance of successful invasion is significantly reduced. Knowing the membrane tension and bending modulus of RBCs it is possible to calculate the relative wrapping energy, which is higher for both Dantu and heterozygous beta thalassaemia individuals (Introini et al., 2022). Using a mechanically realistic model of a deformable RBC and an attractive potential to mimic the adhesion gradients at the surface of the merozoite, Hillringhaus *et al. (* Hillringhaus et al., 2019) found that an increased membrane rigidity results in poor merozoite alignment which could explain why several other RBC polymorphisms associated with membrane tension such as Southeast Asian hereditary ovalocytosis (Mohandas et al., 1984) protect people from malaria infection.

Merozoites were often observed laterally attached to Dantu RBCs but seldom poised for invasion, a recurrent feature observed also in invasion attempts of denser RBCs (Tiffert et al., 2005). Invasion is indeed reduced in dehydrated RBCs (hence higher haemoglobin concentration and cytoplasmatic viscosity), and mutations related to RBC hydration also have effects on RBC deformability (e.g., PIEZO1 (Ma et al., 2018) and the ATP2B4 (Timmann et al., 2012; Lessard et al., 2017; Villegas-Mendez et al., 2021) gene that encodes the plasma membrane calcium ATPase 4 responsible for the intracellular calcium level). PIEZO 1 is a mechanosensitive channel that can be activated when RBCs are stretched in the circulation, causing small-dehydration events *via* brief surges in RBC calcium (Lew and Tiffert, 2017; Rogers and Lew, 2021). An analogous phenomenon may happen during the deformation phase if merozoite contact is sufficient to mechanically activate the PIEZO 1 ion channel. However, studies on the mechanics and susceptibility to invasion of the human gain-of-function PIEZO1 allele variant, E756del, show discordant results (Thye et al., 2022): some did not indicate RBC dehydration or hinder *P. falciparum* invasion and growth *in vitro (* Nguetse et al., 2020), others found that cell density and shear stress contribute to lower parasite replication in E765del PIEZO1 blood *in vitro (* Glushakova et al., 2022). Since the mechanical activation of PIEZO 1 happens when RBCs squeeze within capillaries, future studies under flow might shed light on the role of PIEZO 1 in malaria invasion (see **Section 2**).

### Section 1.4. Merozoite Motility

In general, invasion can be explained as a combined effect of passive processes such as merozoite adhesion-driven wrapping (**Section 1.2**), and active parasite-induced processes such as RBC cytoskeleton remodelling by merozoite ligand injection (Dasgupta et al., 2014) (**Section 1.2**) and merozoite motor contribution (deformations are inhibited when impairing the parasite motor with cytochalasin D) (Weiss et al., 2015; Geoghegan et al., 2021). Merozoites do not have flagella or cilia but instead use a substrate-dependent locomotion called gliding motility to invade target host cells. The motor complex (glideosome) that powers invasion in *Plasmodium* merozoites (Baum et al., 2006) is the same that allows *Plasmodium* sporozoites to traverse tissues, and is also present in tachyzoites of *Toxoplasma gondii* and conserved across other Apicomplexan parasites including *Cryptosporidium (* Wetzel et al., 2005) and *Babesia (* Asada et al., 2012). As a result, this motility mechanism plays a key role in determining the variety of hosts and tissues targeted by these pathogens. Merozoite penetration is driven by the actomyosin motor that drags the tight junction adhesins rearward, thereby creating a traction force that propels the parasite forward into the RBC. The last act is the PVM and host membrane sealing which seems also common among Apicomplexa: *Toxoplasma gondii* shows a twisting (corkscrew-like) motion (Pavlou et al., 2018) and this is probably alike to the helical motility of *Plasmodium* merozoites (Blake et al., 2020; Geoghegan et al., 2021).

*P. falciparum* merozoites were for a long time thought to not display the gliding motility of other stages, but Yahata et al. (Yahata et al., 2021) recently demonstrated that they glide on specific polymer-coated substrates *in vitro*. Gliding behaviour depends on the substrate coating: the percentage of motile parasites on glass coverslip is very low and merozoites follow a Brownian motion when released, this is possibly the reason why this phenomenon has not been observed before with the same microscopy techniques. Tracking merozoite directions after egress with time-lapse imaging shows that *P. falciparum* merozoite average gliding speed is 0.6 μm/s and the longest gliding time is 43 s, significantly lower than *Plasmodium* sporozoites, and *Babesia bovis* merozoites (Yahata et al., 2021). The short duration of *P. falciparum* merozoite motility corresponds to the 2-minute viability of merozoites post egress already shown using optical tweezers (Crick et al., 2014), and indicates declining of motor function over time. It is not clear if this motility is actively used during invasion to discriminate between RBCs, for example based on their mechanical properties, and thus direct invasion in selected cells.

### Section 1.5. Future Perspectives of Biophysical Approaches in Merozoite Invasion

It remains experimentally challenging to investigate how physical traits impact *P. falciparum* invasion due to the short duration of merozoite invasion and the heterogeneity of RBC mechanical properties. Nonetheless, ground-breaking technological improvements are already helping to answer some of the urgent outstanding questions in malaria formulated by Groomes and co-authors (Groomes et al., 2022). Invasion is a balance between parasite and host cell contributions, evolved toward maximal efficient use of parasite force and modulation of the host cell biophysical properties. Merozoite attachment alters the biomechanical properties of the RBC membrane and triggers dramatic surface deformations that are thought to accommodate the parasite for invasion. The mechanism of these large deformation responses remains an open question for future research. A first step forward would be the quantification of the deformation strength based on more standardised physical parameters such as curvature variation and duration, and not only by subjective observation. This can be achieved by continuous advances in imaging technologies (e.g., LLSM) that help reveal novel features of RBC remodelling and clarify concealed mechanisms through previously unachieved resolutions. High-speed video microscopy combined with flickering spectroscopy has played an important role to establish a membrane tension threshold for *Plasmodium* invasion, and in principle it could be optimised to measure the changes in the biophysical properties of the invaded RBC at every step of merozoite reorientation and internalisation. However, flickering spectroscopy relies on the detection of the entire cell contour, and the drastic changes in the RBC membrane during the pre-invasion phase and echinocytosis limit the use of this technique. Finally, if flickering experiments, from video recording to complete analysis, became fully automated and higher throughput, it could be employed to screen compounds that inhibit *P. falciparum* invasion by modifying the host cell membrane biomechanics. If feasible, it should be more difficult for Plasmodium parasites to develop resistance to such a host-targeted approach.

An emerging area of research is the development of fluorophores that allow the measurement of forces (Valanciunaite et al., 2020), and they could be in future applied to the study of RBC membrane biophysics or even food vacuole and endoplasmatic reticulum in living parasites. Probes that are sensitive to lipid order (Niko et al., 2016) and “Flippers” (Goujon et al., 2019) that respond to the change of plasma membrane tension by changing their fluorescence lifetime could be applied to measure lipid organisation and tension of RBCs before and during invasion. However, these techniques rely on Fluorescence Lifetime Imaging Microscopy (FLIM) (Deo, 2020) to measure the probe fluorescence decay time that might be slower than invasion, and therefore their use might be limited by the short duration of malaria invasion.

In malaria, optical tweezers were used directly on RBCs attached to a merozoite to obtain the system detachment force, but optical tweezers have been typically used to quantify the stretchability of cells by trapping and pulling beads conjugated to a target membrane. Future lines of research at the interface between soft matter and cell biology could involve biomimetic approaches to investigate host-pathogen interactions, such as the use of DNA-functionalised colloids of micrometre dimension, vesicles or beads coated with proteins (Mognetti et al., 2019). These synthetic systems provide a better control of ligand adhesion rate and diffusion, overcoming challenges of real biological systems like the wide variability of biophysical properties within a single population of RBCs, even from a single donor. The use of engineered parasites in optical tweezer experiments could help dissecting the orientation of each EBL and Rh families and their contribution to the strength measured.

Finally, future studies should explore how host-pathogen interactions change when the invasion occurs in flow. All studies presented so far have been performed in static cultures, but looking at invasion under flow is important because shear forces can alter ligand-receptor interactions and activate mechanosensitive channels in RBCs. It is known that shaking malaria cultures increases the efficiency of invasion, reduces asynchronous development and multiple invasions (multiple merozoites invading the same RBC) (Allen and Kirk, 2010). This is due to a more uniform distribution of metabolites and nutrients among parasites, as well as a more efficient dispersal of the merozoites after egress (Collins et al., 2017). Shaking can even have a profound effect on the invasion phenotype: *P. falciparum* strains cultured in moving suspension as opposed to static conditions switch from sialic acid-dependent to independent invasion pathways accompanied by upregulation of the corresponding key invasion genes (Awandare et al., 2018; Nyarko et al., 2020). Moving suspension culture may possibly select for parasites with stronger interactions to fasten onto the RBC surface prior to entry, and therefore a thorough study of the hydrodynamic forces at play and how they regulate this switching mechanism will provide insight into the nature of invasion, as occurs *in vivo*. In this view, microfluidic devices and deformation cytometry (Xavier et al., 2016), a technique that relates cell shape to its stiffness based on the chamber flow rate, would be ideal to systematically control the rheology and physical environment while assessing if the familiar features of static invasion, such as RBC deformations, merozoite reorientation and invasion rate, remain the same in flow settings.

## Section 2. Infected Red Blood Cell Mechanics and Cytoadhesion

### Subsection 2.1. Understanding Mechanical Properties of Infected Red Blood Cells With Microfluidic Devices

**Figure 3:**
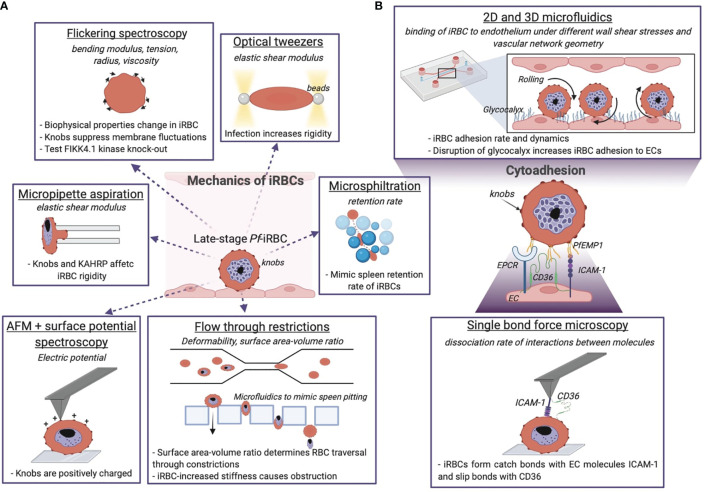
Biophysical methods give insights into the mechanics of *P. falciparum* iRBCs and their cytoadhering features. **(A)** Optical and mechanical techniques have been employed to investigate the effect of the parasite infection onto the host cell membrane biophysics. **(B)** Adhesion between the iRBC and the endothelium is studied at the molecular level and using microfluidic devices, the latter give information about the iRBC dynamics and pathogenesis of malaria by reproducing the physiological *in vivo* situation. The technique is underlined, the quantity measured with the relative technique is in italics, and the dashed sentences report the main results achieved. Image made using ^©^BioRender (https://biorender.com).

A remarkable feature of human RBCs is their high deformability when traversing small diameter capillaries, thus exposing maximal surface area and minimal distance to tissue cells for optimal gas exchange across capillary walls. The principal techniques employed to investigate the biophysical properties of iRBCs described in **Sections 2.1**, **2.2** are summarised in **Figure 3A**. The use of microfluidics has vastly improved our understanding on the mechanical properties of uninfected and iRBC, as well as their interactions with the microvasculature. Microfluidic devices are fabricated by lithography patterning of optically clear flexible polymers, such as poly-dimethylsiloxane (PDMS), and provide control of the size and geometry of microfluidic networks at micrometre resolution (Antia et al., 2008). For example, early studies with microfluidic devices consisting of 2-8 μm microchannel constrictions revealed that *P. falciparum-*infection prevented iRBC passage through small diameter constrictions. Later, wedged PDMS capillary constrictions were used to determine the smallest diameter a cell can pass through without increasing its surface area or lysing. Overall, this study found that schizonts lose the property of flowing through the narrowest constrictions (Herricks et al., 2009) (**Figure 3A**). Moreover, Namvar and colleagues demonstrated using similar chips that surface area-to-volume ratio, not cellular viscoelasticity, determines RBC traversal through small capillaries, the opposite is true for obstruction caused by *P. falciparum-*iRBC (*Pf*-iRBC) trophozoites, which are a result of changes in viscosity and not surface area-to-volume ratio (Namvar et al., 2021).

Late-stage Pf-iRBCs-iRBCs become less deformable and get trapped in the splenic endothelial slits, a narrow 2 μm passage between cell junctions in splenic sinusoids. Microsphiltration assesses the ability of iRBCs to squeeze through narrow fissures between metallic microspheres of 5-25 μm in diameter arranged in a matrix, by computing the retention rate from the upstream and downstream proportions of iRBCs (Deplaine et al., 2011; Lavazec, 2017; Ndour, 2017). It has been applied as high-throughput screening of drug-induced parasite stiffening (Duez et al., 2018). Additionally, microfluidic devices have been used to mimic spleen filtration and the process of pitting with an array of rectangular pillars of PDMS with tuneable slit sizes down to few micrometres (**Figure 3A**) (Rigat-Brugarolas, 2014). Elizalde-Torrent and colleagues showed that by regulating the slit size and the blood flow it is possible to remove the solid parasite from the cytoplasm of an iRBC without destructing the cell (Elizalde-Torrent et al., 2021). Taken together, *Pf-*iRBCs become more rigid as the parasite progresses through the blood developmental cycle, increasing their vulnerability to spleen clearance as shown by the use of microfluidic devices.

### Subsection 2.2. Biophysical Analysis of Infected Red Blood Cell Mechanics

The increase in parasitised RBC rigidity is in part due to the presence of a growing nondeformable intracellular parasite (Fedosov et al., 2011a), and to a number of parasite-encoded proteins, that are exported into the infected cell where they associate with the RBC membrane and cytoskeleton. Plasma membrane deformability of iRBCs has been measured by optical tweezers, as reported in **Figure 3A**, following the thermal motion of single RBCs held in a laser trap (Paul et al., 2019) or by stretching cells attached to beads (Suresh et al., 2005; Mills et al., 2007). The shear modulus of RBCs is found to increase by up to 10-fold during parasite development (Suresh et al., 2005), in the range of 20-60 µN/m at the trophozoite stage and 25-90 µN/m at the schizont stage (Suresh, 2006) (**Table 1**). Another non-invasive optical technique, tomographic phase microscopy, has been used to obtain information about the morphology of parasite vacuoles and decreased haemoglobin content by mapping the refractive index of iRBCs (Park et al., 2008).

A unique feature of *P. falciparum* is sequestration of iRBC in the microvasculature, a survival mechanism to prevent clearance by the spleen and prolong the time in the vasculature to allow the parasite to complete its intra-erythrocytic life cycle. Although sequestration has evolved as a protective mechanism for the parasite, it can lead to severe disease when large numbers of iRBC accumulate in specific microvascular beds. This phenomenon is linked to severe disease complications such as cerebral malaria or placental malaria. Parasite binding to endothelial cells (ECs) is mediated by the variant adhesion protein family *P. falciparum* erythrocyte membrane protein 1 (*Pf*EMP1), which is encoded by a repertoire of approximately 60 *var* genes, and expressed at the surface of the iRBC (Smith et al., 2013). *P. falciparum* has evolved a complex protein machinery and subcellular structures to support trafficking of *Pf*EMP1 to the membrane of iRBC. For example, *P. falciparum* has expanded a family of FIKK kinases implicated in this function. Flickering spectroscopy (**Figure 3A**) seems to suggest that a member of this family, FIKK 4.1, plays also a role in rigidification of the iRBC cytoskeleton (Davies et al., 2020). Other *P. falciparum* erythrocyte-binding proteins such as STEVOR and the ring-infected erythrocyte surface antigen (RESA) have been proved to concur to iRBC membrane rigidity as assessed by microsphere filtrations and ektacytometry (Sanyal et al., 2012). Other parasite features responsible for the rigidification of RBCs are the knobs: 50-150 nm structures formed by the self-assembly of the parasite knob-associated histidine-rich protein (KAHRP). Knobs act as cytoadhesive platforms responsible for the correct display of *Pf*EMP1, while interacting with the host cytoskeleton proteins (Aikawa et al., 1996). KAHRP interactions with the RBC cytoskeleton are highly dynamic; super resolution approaches have recently revealed that it interacts with both β-spectrin and actin in ring stages, while being exclusively associated with actin in trophozoites (Sanchez, 2022). The presence of positively charged knobs partially contributes to iRBC binding to the negative membrane of ECs (Aikawa et al., 1996) (**Figure 3A**), and their increased membrane density in the last 24 hours of parasite maturation has been identified as one of the primary stiffening factors of the host cell (Herricks et al., 2009; Zhang et al., 2015). The role of KAHRP in the increased rigidity of parasitised RBCs has been determined by micropipette aspiration (**Figure 3A**), by measuring the membrane elastic shear modulus of iRBCs. Transgenic *kahrp* knockout parasites presented significant lower membrane shear elastic modulus than wild type lines, and this effect was attributed to the high transcriptional expression of KAHRP and to its clustering at the membrane (Glenister et al., 2002). Recently, the use of flickering spectroscopy by Fröhlich *et al. (* Froühlich et al., 2019) has confirmed iRBC stiffening during the parasite intra-erythrocytic cycle. In particular, trophozoites present a fourfold increase in surface tension increased, and a sevenfold rise in cytosol viscosity compared to uninfected RBCs. Mathematical simulations have revealed that knob formation strongly affects the tension by suppressing membrane fluctuations and increasing membrane-cytoskeleton coupling. In this study, flickering spectroscopy has been also used to better understand how changes in RBCs mechanical properties in hemoglobinopathies might confer protection to severe malaria. Uninfected RBCs containing haemoglobin S (HbAS) and C (HbAC) display surface tensions significantly higher than wild type RBC (HbAA), in line with the idea that higher membrane rigidity hampers parasite infection, and HbAS and HbAC trophozoites show an increased bending modulus and fewer but enlarged knobs (Froühlich et al., 2019).

### Subsection 2.3. Red Blood Cell Interactions With Endothelial Receptors Under Flow

*Pf-*iRBCs bind to a broad array of endothelial receptors. Among these, *Pf*EMP1 binding to ICAM-1 (Ockenhouse et al., 1992) and EPCR (Turner et al., 2013) has been associated to cerebral malaria (Lennartz et al., 2017), binding to CD36 (Barnwell et al., 1989) to uncomplicated malaria (Storm et al., 2019) and binding to the glycan CSA has been linked to placental malaria. **Figure 3B** zooms on iRBCs interactions to endothelial receptors. Early studies of iRBC sequestration under fluid shear stress were performed in bulky flow chambers, usually made of rigid materials impermeable to gas exchange. In these devices, iRBC binding was perfused with a flow pump over a channel seeded with ECs or coated with endothelial receptors. Parasite binding was usually quantified under flow or after fixation of the experiment.

In order to avoid splenic clearance iRBC-EC bonds must withstand considerable forces (2-100 dyne/cm^2^) due to fluid shear stress caused by blood flow (Koutsiaris et al., 2013). There are three types of bonds that can be formed between a receptor-ligand pair: slip bonds, catch bonds, and ideal bonds. Slip bonds break faster when an external force is applied; catch bonds, on the other hand, take longer to dissociate when subjected to an increasing force, and finally the lifetime of an ideal bond is independent on the force acting on it. Lim et al. studied the single molecule interaction between iRBCs and ICAM-1 and CD36 by functionalising an AFM cantilever with these human recombinant proteins (Lim et al., 2017) (**Figure 3B**). They found that indeed ICAM-1 forms catch bond interactions with iRBCs that persist even under flow (single and multiple bonds), while iRBCs formed slip bonds with CD36. Surprisingly the rate of association of iRBC-ICAM-1 bonds is ten times lower than iRBC-CD36 (Lim et al., 2017). This result suggests that ICAM-1 is not the sole mediator for malaria cytoadhesion in the brain, and highlights the importance of other brain receptors such as EPCR.

Over the years, experimenting with the impact of flow has become much more accessible due to the commercialisation of easy to use small single-channel flow chambers. These commercial devices are being used to identify endothelial receptors that mediate binding of *P. falciparum-*tissue culture adapted parasite lines (Lennartz et al., 2017) or field isolates (Storm et al., 2019). These experiments have been crucial to reveal static and rolling interactions with the aforementioned receptors (**Figure 3B**). Flow experiments revealed that the rolling of schizont-stage infected cells under physiological flow conditions resembles the behaviour of leukocytes during acute inflammation (Helms et al., 2016; Dasanna et al., 2017). Mathematical simulations are in agreement with the experimental results (Dasanna et al., 2019), and indicate that the peculiar flip of trophozoites is due to the inertia of the solid parasite mass localised on one side of the host cell (Fedosov et al., 2011b). The rolling behaviour of late stage iRBCs can be linked to knob distribution and the stiffer plasma membrane, which is less prone to elastic deformation in flow (Lansche et al., 2018). Conversely, deformable uninfected RBCs undergo different motions near the vessel wall like tumbling, tank-treading, and rolling depending on the flow rate.

Additionally, microfluidic platforms have proven to be a reliable and physiologically relevant *in vitro* alternative to study the role of endothelial glycocalyx (**Figure 3B**) (Haymet et al., 2021). Glycocalyx breakdown has been recently associated with severe malaria (Hempel et al., 2016). For instance, brain swelling associated to cerebral malaria in children (Seydel et al., 2015), and fatal outcomes in Indonesian adults with *falciparum* malaria (Yeo et al., 2019; Georgiadou and Cunnington, 2019) can be traced to glycocalyx degradation. Nevertheless, the study of glycocalyx on malaria cytoadherence has been hindered by the experimental difficulty to obtain a mature glycocalyx *in vitro (* Barker et al., 2004; Tsvirkun et al., 2017). The glycocalyx participates in sensing mechanotransduction and shear stress influences its structure and thickness (Moore et al., 2021). Glycocalyx thickness varies from hundreds of nm to 1 μm depending on the techniques used to measure it - including electron and confocal microscopy (Kolářová et al., 2014), atomic force microscopy (Marsh and Waugh, 2013), and lately super resolution stochastic optical reconstruction microscopy (STORM) (Fan et al., 2019); and *in vivo* intravital microscopy (Liu et al., 2014; Kataoka et al., 2015). Initial studies on glass microcapillaries coated with a polymer brush to experimentally mimic the glycocalyx layer, found that RBCs travel with a velocity significantly lower than in bare capillaries (Lanotte et al., 2014), leading to the conclusion that the excessive flow resistance found *in vivo* can be attributed to the glycocalyx, as hemodynamic simulations previously suggested (Pries and Secomb, 2005). Perfusion systems lined with a human umbilical vein endothelial cell (HUVEC) monolayer have been instrumental to produce cell alignment (Sinha et al., 2016) and a glycocalyx layer similar to the one seen *in vivo (* Gouverneur et al., 2006). The loss of the sialic acids of the EC glycocalyx by enzymatic treatment increases adhesion of iRBCs without affecting their motion in proximity to the endothelium but decreasing their velocity by 10-14% (Introini et al., 2018b).

### Subsection 2.4. Bioengineered 3D Microvessel Models to Study Malaria Pathogenesis

A main limitation of flow chambers is that they generally consist of a 1 mm-wide single channel in a linear display, and are therefore far away from recapitulating the complex hierarchical microvascular geometry or small vascular constrictions such as 5-10 μm capillaries or 2 μm endothelial slits. Recently, brain organoids have emerged as important 3D multicellular models (Velasco et al., 2019; Adams, 2021), but they have very heterogeneous non-controllable architecture and until today these systems lack perfusable vasculature.

Bioengineered 3D microvessel models are physiologically relevant alternatives to understand vascular dysfunctions and infections, as they reproduce the microvasculature architecture, geometry, and fluidic properties (Tan et al., 2020). These devices are built using microfabrication techniques, in hydrogels such as collagen or fibrin enclosed in PDMS or acrylic housing jigs. The dimensions of the endothelial microvessel networks can be controlled with micrometre precision by soft lithography patterning (Long et al., 2021). Bernabeu et al. examined the binding affinity of *Pf-*iRBC to 3D brain microvessels along a shear gradient (Bernabeu et al., 2019). This revealed that infected cells expressing *Pf*EMP1 that bind to ICAM-1 were more adherent at shear forces greater than 1 dyne/cm^2^, further demonstrating iRBC-ICAM-1 catch bond behaviour. Binding of iRBC to EPCR was enhanced under low shear stress, suggesting that this bond might display catch-bond behaviour at lower shear forces (Bernabeu et al., 2019). However, future AFM approaches are needed to confirm this finding.

Further advances in microfabrication have generated endothelialised capillary-size vessels down to 5 μm in diameter by using multiphoton ablation (Rayner et al., 2021). This cutting-edge approach was used to study malaria microvascular obstruction and revealed that the rigidity of *Pf-*iRBC alone is not responsible for capillary blockade (Arakawa et al., 2020). Knobless parasites expressing *Pf*EMP1 accumulated in the boundary between capillaries and venules, and the presence of knobs increased the affinity to capillary regions exposed to high shear flow. This system revealed highly distinctive spatial and temporal kinetics of iRBC accumulation and reproduced sequestration patterns observed in fatal cerebral malaria patients (Milner et al., 2015).

The study of malaria pathogenesis in more physiological systems is important, as endothelial transcription is modulated by both flow and stiffness of the extracellular matrix substrate. ECs possess mechanosensitive molecules such as caveolins, PIEZO1 or the glycocalyx that activate transcriptional pathways that modulate receptor expression as well as junctional proteins in response to culture under flow (Raasch et al., 2015; Reinitz et al., 2015). Furthermore, ECs can sense the mechanics of the surrounding tissue *via* the formation of focal adhesions (Wang et al., 2019), and when grown on stiff substrates become more permeable and sensitive to barrier disruptive molecules (Gordon et al., 2020). For example, ECs cultured on stiffer substrates have reduced glycocalyx (Mahmoud et al., 2021) and ICAM-1 (Scott et al., 2016) expression. Collectively this implies that the mechanics of the extracellular matrix may impact endothelial function and malaria pathology. While no studies to date have directly compared the effect of substrate stiffness to iRBC binding, future research needs to take these variables into account, as *Pf-*iRBC sequestration in the microvasculature of humans occurs at different stiffnesses (Zhang and Reinhart-King, 2020) and under a wide range of flow properties. As highlighted in **Figure 3B**, the study of malaria pathogenesis in bioengineered 3D microvessels with tunable flow and substrate biomechanical properties offers the opportunity to answer these questions.

### Subsection 2.5. Future Perspective on Biophysics of Red Blood Cell Interactions With Host Cells

Microfluidics is essential for the study of cytoadhesion because it allows systematic tuning of biological (haematocrit, parasitaemia) and physical (flow rate, geometry, substrate stiffness, temperature) parameters (Krishnan et al., 2011), however many aspects need to be explored further. For example, malaria cytoadherence increases at an elevated temperature (Udomsangpetch et al., 2002; Zhang et al., 2018), while deformability of trophozoites and schizonts decreases irreversibly (Mills et al., 2007; Park et al., 2008), and this plastic behaviour of iRBCs in response to fever could have implications when treatments are used to suppress fever episodes. The possibility of adopting customised designs and materials that reflect a range of vascular organisations, architectures, and cellular compositions across different organs (Budday et al., 2015) could shed light on the heterogeneity of malaria pathology. With the support of computational modelling (Lykov et al., 2015; Ishida et al., 2017), it could deliver a comprehensive picture of malaria disease to direct drug intervention. Another important bottleneck of current imaging experiments, both static and in flow, is image analysis. This is currently done almost exclusively manually, selecting individual cells, and following their behaviour over time. There is an urgent need for automated image analysis methods that can handle large image datasets in different conditions and extract key cell properties. This advancement would improve experiment reproducibility and provide more quantitative information.

An important host response to malaria infection is the activation of multiple immune pathways to facilitate parasite clearance. However, the subsequent release of cytokines can activate the endothelium, promote iRBC sequestration and severity (Pais and Penha-Gonçalves, 2018). While these immunological interactions play crucial roles in influencing malaria pathogenesis, the biomechanical processes that underpin them remain unknown. Instead, the biomechanics of phagocytosis and immune synapse formation have been well studied in the context of other diseases, as reviewed in (Chua et al., 2021). Monocytes, macrophages and neutrophils have all demonstrated various abilities to phagocytose *Pf*-iRBC and merozoites. Phagocytes recognise target cells, particles, or debris which stimulates the cell to form a cup-like protrusion that spreads over the target and resulting in its eventual engulfment. The spatiotemporal forces that are exerted on a particle during phagocytosis have recently been determined by microparticle traction force microscopy and LLSM, revealing that macrophages generate F-actin and myosin-I rich “teeth”-like protrusions (Vorselen et al., 2021). Interestingly, macrophages were three times more efficient at phagocytosing stiffened microparticles (Jaumouillé et al., 2019), and need longer period of time to internalise ellipsoid particles over spherical ones (Paul et al., 2013). It is therefore likely that significantly stiffer late stage iRBCs are more easily phagocytosed during infection. Likewise, CD8+ T cells and natural killer cells show greater activation (Liu et al., 2021), form larger synapses (Friedman et al., 2021), and demonstrate greater cytotoxicity against stiffer targets (Tello-Lafoz et al., 2021). Critically, cytotoxic T cells and macrophages accumulate in the cerebral vasculature during cerebral malaria, and are thought to be at least partially responsible for disease severity (Riggle et al., 2020). Understanding the biomechanics and the functional consequences of iRBC interactions with the human immune system in the microvascular context will shed light on malaria pathogenesis.

## Section 3. Malaria Species Beyond *Plasmodium falciparum*

Only five out of over 200 known *Plasmodium* species are clinically relevant to humans: *P. falciparum, P. vivax, P. malariae, P. ovale, and P. knowlesi*. Of these, *P. falciparum* and *P. vivax* contribute the majority of cases. *P. falciparum* remains the only species for which all stages have been cultured *in vitro*, and most research efforts and antimalarial therapies/vaccine candidates are focused on it. The lack of research in other species, particularly *P. vivax* and the zoonotic species *P. knowlesi*, will be the limiting factor that will hold malaria elimination back. These species are quite distantly related to each other, and rely on both unique and conserved molecular interactions to invade human cells and cause a broad spectrum of clinical presentations, posing quite different challenges for disease control and antimalarial development (Keeling and Rayner, 2015). For example, *P. vivax* can remain in dormant forms in the liver causing a relapse of the disease at a later time (Prudencio et al., 2006).

*Plasmodium* species have different durations of the intraerythrocytic cycle, ranging from 27 (*P. knowlesi*), 48 (*P. falciparum, vivax, and ovale*), and 72 hours (*P. malariae*), and number of daughter merozoites in a schizont (up to 16 for *P. knowlesi* and to 24 for *P. vivax*). *P. knowlesi* merozoites are 2-3 μm, twice the size of their *falciparum* counterparts, and therefore easier to follow by video microscopy. The first images of malaria merozoites were recorded in 1975 by Dvorak et al. (Dvorak et al., 1975) were in fact of *P. knowlesi* infecting monkey RBCs. Thanks to their larger dimensions and by using *P. knowlesi* merozoites expressing fluorescent-tagged apical membrane antigen-1 (AMA-1), it was demonstrated for the first time that the apical complex is located at the wider end of the zoite (Yahata et al., 2021), contrary to what previously thought from early images of invading parasites by transmission electron microscopy (Miller et al., 1979). *P. knowlesi* have longer-lived (316 s) merozoites that glide onto RBC surfaces at 1.1 μm/s, faster than *P. falciparum*. Moreover, they can glide across multiple human RBC, up to 7, before selecting a cell to invade, and this behaviour resembles the travel of sporozoites along several hepatocytes before invading one (Mota et al., 2001; Tavares et al., 2013). Gliding could be advantageous for the parasites to sense suitable RBCs for invasion, facilitating the identification of preferred cells like young RBCs called reticulocytes which make up for less than 2% of circulating RBCs.

Mechanical and rheological differences among *Plasmodium* species are of pathological relevance. *P. vivax* has a distinct preference for immature reticulocytes, identified by the transferrin receptor (CD71) on their surface that is progressively lost during reticulocyte maturation into erythrocyte. Invasion accelerates the ejection of host material, including CD71, completing the maturation by 3 hours post invasion (Malleret, 2015). Similarly to *P. falciparum (* Narla and Mohandas, 2017)*, P. knowlesi*-infected human RBCs (Barber et al., 2018) become more rigid progressing throughout the blood stage. Instead, both bulk and single-cell techniques (micropipette aspiration, laminar shear flow system, microfluidics) show that *P. vivax*-rings are more deformable than uninfected cells (Suwanarusk et al., 2004; Handayani et al., 2009; Malleret, 2015). This is possibly related to a two-fold increase in surface area of *Pv*-iRBCs, and hence a marked increase in their surface area to volume ratio (Suwanarusk et al., 2004). Methods to measure the RBC mechanical properties can assist in establishing whether higher RBC tension confers protection across red cell ages and genetic backgrounds, and explore whether *Plasmodium* species and strains are differently affected by this mechanism. This will be critical to understanding whether tension is a potential cross-species intervention target.

Cytoadherence phenomenon was long believed to be restricted to *P. falciparum* infection, however some evidence now supports that also other *Plasmodium* species undergo cytoadhesion events (Totino and Lopes, 2017). The occurrence of severe forms of *P. vivax* infections, such as cerebral and placental malaria, which were previously reported to be exclusively associated with *P. falciparum*, suggests that *P. vivax* can, to some extent, present adhesive phenotypes, even if it does not present any protein homologous to *Pf*EMP1 *(* Totino and Lopes, 2017). Rosette formation, when uninfected RBCs adhere to iRBCs, appears in *P. falciparum*, *P. vivax (* Lee et al., 2014)*, and P. knowlesi*, even though in the latter with less frequency and in the presence of human serum (Lee et al., 2021). Knob formation and sequestration are distinct characteristics of Pf-iRBCs. Pk-iRBCs *(* Russell and Cooke, 2017) do not develop knobs, but still can bind to lung and kidney endothelial cells primed with *P. knowlesi* culture supernatant *via* a functional ortholog of *Pf*EMP1, although not to the cerebral endothelium (Lee et al., 2021). Such novel findings on the capability of other *Plasmodium* species to undergo cytoadhesion beyond *P. falciparum* will require further investigations that can be addressed by the use of biophysical approaches and concepts.

## Concluding Remarks

In this review we have emphasised the state-of-the-art technologies developed to probe the mechanics of malaria infection, specifically during *Plasmodium* invasion, maturation, and cytoadhesion, and how these would shape the future directions of malaria research. The contribution of RBC mechanics to invasion and vascular sequestration has recently become more evident thanks to the numerous biophysical techniques developed and applied to malaria. Some methods from soft matter, surface physics, and biomimetic nanotechnology (Wei et al., 2022) have been implemented to develop alternative therapeutic strategies, and together with novel ideas from the physics area that can be optimised for malaria research (Mognetti et al., 2019; Saint-Sardos et al., 2020) they should become part of our future toolbox to tackle new research questions.

## Author Contributions

VI, MG, and MB wrote the manuscript. JR, PC, and MB provided feedback and corrected the manuscript. All authors contributed to the article and approved the submitted version.

## Funding

VI is funded by the Wellcome Trust Junior Interdisciplinary Fellowship (Wellcome 20485/Z/16/Z). MG is funded by the EIPOD4 fellowship programme (Grant agreement number 847543). PC and his lab are funded by the EPSRC (EP/R011443/1). JR and his lab are funded by the Wellcome Trust (220266/Z/20/Z). MB and her lab receive core program funding of the European Molecular Biology Laboratory (EMBL).

## Conflict of Interest

The authors declare that the research was conducted in the absence of any commercial or financial relationships that could be construed as a potential conflict of interest.

## Publisher’s Note

All claims expressed in this article are solely those of the authors and do not necessarily represent those of their affiliated organizations, or those of the publisher, the editors and the reviewers. Any product that may be evaluated in this article, or claim that may be made by its manufacturer, is not guaranteed or endorsed by the publisher.

## References

[B1] Abdalrahman T. Franz T. (2017). Analytical Modeling of the Mechanics of Early Invasion of a Merozoite Into a Human Erythrocyte. J. Biol. Phys. 43 (4), 471–479. doi: 10.1007/s10867-017-9463-6 28914402 PMC5696301

[B2] Abkarian M. Massiera G. Berry L. Roques M. Braun-Breton C. (2011). A Novel Mechanism for Egress of Malarial Parasites From Red Blood Cells. Blood 117 (15), 4118–4124. doi: 10.1182/blood-2010-08-299883 21297002

[B3] Adams Y. Olsen R. W. Bengtsson A. Dalgaard N. Zdioruk M. Satpathi S. Behera P. K.. (2021). *Plasmodium Falciparum* Erythrocyte Membrane Protein 1 Variants Induce Cell Swelling and Disrupt the Blood-Brain Barrier in Cerebral Malaria. J. Exp. Med. 218 (3), e20201266. doi: 10.1084/jem.20201266 33492344 PMC7833209

[B4] Ademiloye A. S. Zhang L. W. Liew K. M. (2017). A Three-Dimensional Quasicontinuum Approach for Predicting Biomechanical Properties of Malaria-Infected Red Blood Cell Membrane. Appl. Math. Modelling 49, 35–47. doi: 10.1016/j.apm.2017.04.030

[B5] Aikawa M. (1997). Studies on *Falciparum* Malaria With Atomic-Force and Surface-Potential Microscopes. Ann. Trop. Med. Parasitol. 91 (7), 689–692. doi: 10.1080/00034989760419 9625922

[B6] Aikawa M. Kamanura K. Shiraishi S. Matsumoto Y. Arwati H. Torii M. (1996). Membrane Knobs of Unfixed *Plasmodium Falciparum* Infected Erythrocytes: New Findings as Revealed by Atomic Force Microscopy and Surface Potential Spectroscopy. Exp. Parasitol. 84 (3), 339–343. doi: 10.1006/expr.1996.0122 8948323

[B7] Akaki M. Nagayasu E. Nakano Y. Aikawa M. (2002). Surface Charge of *Plasmodium Falciparum* Merozoites as Revealed by Atomic Force Microscopy With Surface Potential Spectroscopy. Parasitol. Res. 88 (1), 16–20. doi: 10.1007/s004360100485 11822732

[B8] Allen R. J. W. Kirk K. (2010). *Plasmodium Falciparum* Culture: The Benefits of Shaking. Mol. Biochem. Parasitol. 169 (1), 63–65. doi: 10.1016/j.molbiopara.2009.09.005 19766147

[B9] Amino R. Thiberge S. Martin B. Celli S. Shorte S. Frischknecht F. (2006). Quantitative Imaging of *Plasmodium* Transmission From Mosquito to Mammal. Nat. Med. 12 (2), 220–224. doi: 10.1038/nm1350 16429144

[B10] Aniweh Y. Gao X. Hao P. Meng W. Lai S. K. Gunalan K. (2017). *P. Falciparum* RH5-Basigin Interaction Induces Changes in the Cytoskeleton of the Host RBC. Cell. Microbiol. 19 (9), e12747. doi: 10.1111/cmi.12747 28409866

[B11] Antia M. Gao X. Hao P. Meng W. Lai S. K. Gunalan K. (2008). Microfluidic Approaches to Malaria Pathogenesis. Cell. Microbiol. 10 (10), 1968–1974. doi: 10.1111/j.1462-5822.2008.01216.x 18754851 PMC3818003

[B12] Arakawa C. Gunnarsson C. Howard C. Bernabeu M. Phong K. Yang E. (2020). Biophysical and Biomolecular Interactions of Malaria-Infected Erythrocytes in Engineered Human Capillaries. Sci. Adv. 6 (3), eaay7243. doi: 10.1126/sciadv.aay7243 32010773 PMC6968943

[B13] Asada M. Goto Y. Yahata K. Yokoyama N. Kawai S. Inoue N. (2012). Gliding Motility of *Babesia Bovis* Merozoites Visualized by Time-Lapse Video Microscopy. PloS One 7 (4), e35227. doi: 10.1371/journal.pone.0035227 22506073 PMC3323635

[B14] Awandare G. A. Nyarko P. B. Aniweh Y. Ayivor-Djanie R. Stoute J. A. (2018). *Plasmodium Falciparum* Strains Spontaneously Switch Invasion Phenotype in Suspension Culture. Sci. Rep. 8 (1), 5782. doi: 10.1038/s41598-018-24218-0 29636510 PMC5893586

[B15] Bannister L. H. Mitchell G. H. Butcher G. A. Dennis E. D. Cohen S. (1986). Structure and Development of the Surface Coat of Erythrocytic Merozoites of *Plasmodium Knowlesi* . Cell Tissue Res. 245 (2), 281–290. doi: 10.1007/bf00213933 3742563

[B16] Barber B. E. Russell B. Grigg M. J. Zhang R. William T. Amir A. (2018). Reduced Red Blood Cell Deformability in *Plasmodium Knowlesi* Malaria. Blood Adv. 2 (4), 433–443. doi: 10.1182/bloodadvances.2017013730 29487058 PMC5858481

[B17] Barker A. L. Konopatskaya O. Neal C. R. Macpherson J. V. Whatmore J. L. Winlove C. P. (2004). Observation and Characterisation of the Glycocalyx of Viable Human Endothelial Cells Using Confocal Laser Scanning Microscopy. Phys. Chem. Chem. Phys. 6, 1006–1011. doi: 10.1039/B312189E

[B18] Barnwell J. W. Asch A. S. Nachman R. L. Yamaya M. Aikawa M. Ingravallo P. (1989). A Human 88-kD Membrane Glycoprotein (CD36) Functions *In Vitro* as a Receptor for a Cytoadherence Ligand on *Plasmodium Falciparum*-Infected Erythrocytes. J. Clin. Invest. 84 (3), 765–772. doi: 10.1172/JCI114234 2474574 PMC329717

[B19] Baum J. Papenfuss A. T. Baum B. Speed T. P. Cowman A. F. (2006). Regulation of Apicomplexan Actin-Based Motility. Nat. Rev. Microbiol. 4 (8), 621–628. doi: 10.1038/nrmicro1465 16845432

[B20] Bernabeu M. Gunnarsson C. Vishnyakova M. Howard C. C. Nagao R. J. Avril M. (2019). Binding Heterogeneity of *Plasmodium Falciparum* to Engineered 3D Brain Microvessels Is Mediated by EPCR and ICAM-1. MBio 10 (3), e00420–e00419. doi: 10.1128/mBio.00420-19 31138740 PMC6538777

[B21] Bessis M. Mohandas N. Feo C. (1980). Automated Ektacytometry: A New Method of Measuring Red Cell Deformability and Red Cell Indices. Blood Cells 6 (3), 315–327. doi: 10.1007/978-3-642-67756-4_13 7397390

[B22] Betz T. Lenz M. Joanny J. F. Sykes C. (2009). ATP-Dependent Mechanics of Red Blood Cells. PNAS 106 (36), 15320–15325. doi: 10.1073/pnas.0904614106 19717437 PMC2741249

[B23] Blake T. C. A. Haase S. Baum J. (2020). Actomyosin Forces and the Energetics of Red Blood Cell Invasion by the Malaria Parasite *Plasmodium Falciparum* . PloS Pathog. 16 (10), e1009007. doi: 10.1371/journal.ppat.1009007 33104759 PMC7644091

[B24] Braun-Breton C. Abkarian M. (2016). Red Blood Cell Spectrin Skeleton in the Spotlight. Trends Parasitol. 32 (2), 90–92. doi: 10.1016/j.pt.2015.11.011 26652974

[B25] Budday S. Nay R. de Rooij R. Steinmann P. Wyrobek T. Ovaert T. C. (2015). Mechanical Properties of Gray and White Matter Brain Tissue by Indentation. J. Mechanical Behav. Biomed. Mater. 46, 318–330. doi: 10.1016/j.jmbbm.2015.02.024 PMC4395547 25819199

[B26] Bustamante L. Y. Powell G. T. Lin Y. C. Macklin M. D. Cross N. Kemp A. (2017). Synergistic Malaria Vaccine Combinations Identified by Systematic Antigen Screening. PNAS 114 (45), 12045–12050. doi: 10.1073/pnas.1702944114 29078270 PMC5692528

[B27] Byeon H. Ha Y. R. Lee S. J. (2015). Holographic Analysis on Deformation and Restoration of Malaria-Infected Red Blood Cells by Antimalarial Drug. J. Biomed. Optics 20 (11), 115003. doi: 10.1117/1.JBO.20.11.115003 26544670

[B28] Callan-Jones A. Albarran Arriagada O. E. Massiera G. Lorman V. Abkarian M. (2012). Red Blood Cell Membrane Dynamics During Malaria Parasite Egress. Biophys. J. 103 (12), 2475–2483. doi: 10.1016/j.bpj.2012.11.008 23260049 PMC3525858

[B29] Chasis J. A. Mohandas N. Shohet S. B. (1985). Erythrocyte Membrane Rigidity Induced by Glycophorin A-Ligand Interaction. Evidence for a Ligand-Induced Association Between Glycophorin A and Skeletal Proteins. J. Clin. Invest. 75 (6), 1919–1926. doi: 10.1172/JCI111907 4008645 PMC425549

[B30] Chen B. C. Legant W. R. Wang K. Shao L. Milkie D. E. Davidson M. W. (2014). Lattice Light-Sheet Microscopy: Imaging Molecules to Embryos at High Spatiotemporal Resolution. Science 346 (6208), 1257998. doi: 10.1126/science.1257998 25342811 PMC4336192

[B31] Cho S. Kim S. Kim Y. Park Y. (2012). Optical Imaging Techniques for the Study of Malaria. Trends Biotechnol. 30 (2), 71–79. doi: 10.1016/j.tibtech.2011.08.004 21930322

[B32] Chua C. L. L. Ng I. M. J. Yap B. J. M. Teo A. (2021). Factors Influencing Phagocytosis of Malaria Parasites: The Story So Far. Malaria J. 20 (1), 319. doi: 10.1186/s12936-021-03849-1 PMC8284020 34271941

[B33] Collins C. R. Hackett F. Atid J. Tan M. S. Y. Blackman M. J. (2017). The *Plasmodium Falciparum* Pseudoprotease SERA5 Regulates the Kinetics and Efficiency of Malaria Parasite Egress From Host Erythrocytes. PloS Pathog. 13 (7), e1006453. doi: 10.1371/journal.ppat.1006453 28683142 PMC5500368

[B34] Cowman A. F. Tonkin C. J. Tham W. H. Duraisingh M. T. (2017). The Molecular Basis of Erythrocyte Invasion by Malaria Parasites. Cell Host Microbe 22 (2), 232–245. doi: 10.1016/j.chom.2017.07.003 28799908 PMC12801281

[B35] Crick A. J. Tiffert T. Shah S. M. Kotar J. Lew V. L. Cicuta P. (2013). An Automated Live Imaging Platform for Studying Merozoite Egress-Invasion in Malaria Cultures. Biophys. J. 104 (5), 997–1005. doi: 10.1016/j.bpj.2013.01.018 23473482 PMC3870799

[B36] Crick A. J. Theron M. Tiffert T. Lew V. L. Cicuta P. Rayner J. C. (2014). Quantitation of Malaria Parasite-Erythrocyte Cell-Cell Interactions Using Optical Tweezers. Biophys. J. 107 (4), 846–853. doi: 10.1016/j.bpj.2014.07.010 25140419 PMC4142257

[B37] Crosnier C. Bustamante L. Y. Bartholdson S. J. Bei A. K. Theron M. Uchikawa M. (2011). Basigin Is a Receptor Essential for Erythrocyte Invasion by *Plasmodium Falciparum* . Nature 480, 534–537. doi: 10.1038/nature10606 22080952 PMC3245779

[B38] Dasanna A. K. Lansche C. Lanzer M. Schwarz U. S. (2017). Rolling Adhesion of Schizont Stage Malaria-Infected Red Blood Cells in Shear Flow. Biophys. J. 112 (9), 1908–1919. doi: 10.1016/j.bpj.2017.04.001 28494961 PMC5425411

[B39] Dasanna A. K. Fedosov D. A. Gompper G. Schwarz U.S. (2019). State Diagram for Wall Adhesion of Red Blood Cells in Shear Flow: From Crawling to Flipping. Soft. Matter 15 (27), 5511–5520. doi: 10.1039/c9sm00677j 31241632

[B40] Dasgupta S. Auth T. Gov N. S. Satchwell T. J. Hanssen E. Zuccala E. S. (2014). Membrane-Wrapping Contributions to Malaria Parasite Invasion of the Human Erythrocyte. Biophys. J. 107 (1), 43–54. doi: 10.1016/j.bpj.2014.05.024 24988340 PMC4184798

[B41] Davidson M. S. Andradi-Brown C. Yahiya S. Chmielewski J. O’Donnell A. J. Gurung P. (2021). Automated Detection and Staging of Malaria Parasites From Cytological Smears Using Convolutional Neural Networks. Biol. Imaging 1, e2. doi: 10.1017/S2633903X21000015 35036920 PMC8724263

[B42] Davies H. Belda H. Broncel M. Ye X. Bisson C. Introini V. (2020). An Exported Kinase Family Mediates Species-Specific Erythrocyte Remodelling and Virulence in Human Malaria. Nat. Microbiol. 5 (6), 848–863. doi: 10.1038/s41564-020-0702-4 32284562 PMC7116245

[B43] Deo C. (2020). Hybrid Fluorescent Probes for Imaging Membrane Tension Inside Living Cells. ACS Cent. Sci. 6 (8), 1285–1287. doi: 10.1021/acscentsci.0c00977 32875071 PMC7453413

[B44] Deplaine G. Safeukui I. Jeddi F. Lacoste F. Brousse V. Perrot S. (2011). The Sensing of Poorly Deformable Red Blood Cells by the Human Spleen Can Be Mimicked. Vitro Blood 117 (8), e88–e95. doi: 10.1182/blood-2010-10-312801 21163923 PMC3062417

[B45] Depond M. Henry B. Buffet P. Ndour P. A. (2019). Methods to Investigate the Deformability of RBC During Malaria. Front. Physiol. 10. doi: 10.3389/fphys.2019.01613 PMC6990122 32038293

[B46] Duez J. Carucci M. Garcia-Barbazan I. Corral M. Perez O. Presa J. L. (2018). High-Throughput Microsphiltration to Assess Red Blood Cell Deformability and Screen for Malaria Transmission–Blocking Drugs. Nat. Protoc. 13 (6), 1362–1376. doi: 10.1038/nprot.2018.035 29844524

[B47] Dulinska I. Targosz M. Strojny W. Lekka M. Czuba P. Balwierz W. (2006). Stiffness of Normal and Pathological Erythrocytes Studied by Means of Atomic Force Microscopy. J. Biochem. Biophys. Methods 66 (1-3), 1–11. doi: 10.1016/j.jbbm.2005.11.003 16443279

[B48] Dvorak J. A. Miller L. H. Whitehouse W. C. Shiroishi T. (1975). Invasion of Erythrocytes by Malaria Merozoites. Science 187 (4178), 748–750. doi: 10.1126/science.803712 803712

[B49] Ebel E. R. Kuypers F. A. Lin C. Petrov D. A. Egan E. S. (2021). Common Host Variation Drives Malaria Parasite Fitness in Healthy Human Red Cells. ELife 10, e69808. doi: 10.7554/eLife.69808 34553687 PMC8497061

[B50] Elizalde-Torrent A. Trejo-Soto C. Méndez-Mora L. Nicolau M. Ezama O. Gualdrón-López M. (2021). Pitting of Malaria Parasites in Microfluidic Devices Mimicking Spleen Interendothelial Slits. Sci. Rep. 11 (1), 22099. doi: 10.1038/s41598-021-01568-w 34764379 PMC8585870

[B51] Engelhardt H. Gaub H. Sackmann E. (1984). Viscoelastic Properties of Erythrocyte Membranes in High-Frequency Electric Fields. Nature 30, 7378. doi: 10.1038/307378a0 6694733

[B52] Esposito A. Choimet J. B. Skepper J. N. Mauritz J. M.A. Lew V. L. Kaminski C. F. (2010). Quantitative Imaging of Human Red Blood Cells Infected With *Plasmodium Falciparum* . Biophys. J. 99 (3), 953–960. doi: 10.1016/j.bpj.2010.04.065 20682274 PMC2913174

[B53] Evans E. Mohandas N. Leung A. (1984). Static and Dynamic Rigidities of Normal and Sickle Erythrocytes. Major Influence of Cell Hemoglobin Concentration. J. Clin. Invest. 73 (2), 477–488. doi: 10.1172/jci111234 6699172 PMC425039

[B54] Evans J. Gratzer W. Mohandas N. Parker Sleep K. J. (2008). Fluctuations of the Red Blood Cell Membrane: Relation to Mechanical Properties and Lack of ATP Dependence. Biophys. J. 94 (10), 4134–4144. doi: 10.1529/biophysj.107.117952 18234829 PMC2367166

[B55] Fan J. Sun Y. Xia Y. Tarbell J. M. Fu B. M. (2019). Endothelial Surface Glycocalyx (ESG) Components and Ultra-Structure Revealed by Stochastic Optical Reconstruction Microscopy (STORM). Biorheology 56 (2-3), 77–88. doi: 10.3233/BIR-180204 31045510

[B56] Fedosov D. A. Caswell B. Suresh S. Karniadakis G. E. (2011a). Quantifying the Biophysical Characteristics of *Plasmodium-Falciparum*-Parasitized Red Blood Cells in Microcirculation. PNAS 108 (1), 35–39. doi: 10.1073/pnas.1009492108 21173269 PMC3017137

[B57] Fedosov D. A. Caswell B. Karniadakis G. E. (2011b). Wall Shear Stress-Based Model for Adhesive Dynamics of Red Blood Cells in Malaria. Biophys. J. 100 (9), 2084–2093. doi: 10.1016/j.bpj.2011.03.027 21539775 PMC3149244

[B58] Fontes A. Fernandes H. P. de Thomaz A. A. Barbosa L. C. Barjas-Castro M. L. Cesar C. L. (2008). Measuring Electrical and Mechanical Properties of Red Blood Cells With Double Optical Tweezers. J. Biomed. Optics 13 (1), 014001. doi: 10.1117/1.2870108 18315359

[B59] Frevert U. Engelmann S. Zougbédé S. Stange J. Ng B. Matuschewski K. Liebes L. (2005). Intravital Observation of *Plasmodium Berghei* Sporozoite Infection of the Liver. PloS Biol. 3, e192. doi: 10.1371/journal.pbio.0030192 15901208 PMC1135295

[B60] Friedman D. Simmonds P. Hale A. Bere L. Hodson N. W. White M. R. H. (2021). Natural Killer Cell Immune Synapse Formation and Cytotoxicity are Controlled by Tension of the Target Interface. J. Cell Sci. 134 (7), jcs.258570. doi: 10.1242/jcs.258570 PMC8077183 33712452

[B61] Frischknecht F. Matuschewski K. (2017). *Plasmodium* Sporozoite Biology. Cold Spring Harbor Perspect. Med. 7, a025478. doi: 10.1101/cshperspect.a025478 PMC5411682 28108531

[B62] Froühlich B. Jäger J. Lansche C. Sanchez C. P. Cyrklaff M. Buchholz B. (2019). Hemoglobin S and C Affect Biomechanical Membrane Properties of *P. Falciparum* -Infected Erythrocytes. Commun. Biol. 2 (1), 1–11. doi: 10.1038/s42003-019-0556-6 31428699 PMC6692299

[B63] Geoghegan N. D. Evelyn C. Whitehead L. W. Pasternak M. McDonald P. Triglia T. (2021). 4D Analysis of Malaria Parasite Invasion Offers Insights Into Erythrocyte Membrane Remodeling and Parasitophorous Vacuole Formation. Nat. Commun. 12, 3620. doi: 10.1038/s41467-021-23626-7 34131147 PMC8206130

[B64] Georgiadou A. Cunnington A. J. (2019). Shedding of the Vascular Endothelial Glycocalyx: A Common Pathway to Severe Malaria? Clin. Infect. Dis.: Off. Publ. Infect. Dis. Soc. America 69 (10), 1721–1723. doi: 10.1093/cid/ciz043 30698670

[B65] Gilson P. R. Crabb B. S. (2009). Morphology and Kinetics of the Three Distinct Phases of Red Blood Cell Invasion by *Plasmodium Falciparum* Merozoites. Int. J. Parasitol. 39 (1), 91–96. doi: 10.1016/j.ijpara.2008.09.007 18952091

[B66] Glenister F. K. Coppel R. L. Cowman A. F. Mohandas N. Cooke B. M. (2002). Contribution of Parasite Proteins to Altered Mechanical Properties of Malaria-Infected Red Blood Cells. Blood 1 99 (3), 1060–1063. doi: 10.1182/blood.v99.3.1060 11807013

[B67] Glodek A. M. Mirchev R. Golan D. E. Khoory J. A. Burns J. M. Shevkoplyas S. S. (2010). Ligation of Complement Receptor 1 Increases Erythrocyte Membrane Deformability. Blood 116 (26), 6063–6071. doi: 10.1182/blood-2010-04-273904 20861458 PMC3031392

[B68] Glushakova S. Yin D. Li T. Zimmerberg J. (2005). Membrane Transformation During Malaria Parasite Release From Human Red Blood Cells. Curr. Biol. 15 (18), 1645–1650. doi: 10.1016/j.cub.2005.07.067 16169486

[B69] Glushakova S. Kegawa Y. Bezrukov L. Waters H. Blank P. S. Zimmerberg J. (2022). PIEZO1 E756del Gain of Function Mutation Makes Human Erythrocytes Less Habitable for Malaria Parasites. Biophys. J. 121 (3), 495a. doi: 10.1016/j.bpj.2021.11.332

[B70] Gordon E. Schimmel L. Frye M. (2020). The Importance of Mechanical Forces for *In Vitro* Endothelial Cell Biology. Front. Physiol. 11. doi: 10.3389/fphys.2020.00684 PMC7314997 32625119

[B71] Goujon A. Colom A. Straková K. Mercier V. Mahecic D. Manley S. (2019). Mechanosensitive Fluorescent Probes to Image Membrane Tension in Mitochondria, Endoplasmic Reticulum, and Lysosomes. J. Am. Chem. Soc. 141 (8), 3380–3384. doi: 10.1021/jacs.8b13189 30744381

[B72] Gouverneur M. Spaan J. A.E. Pannekoek H. Fontijn R. D. Vink H. (2006). Fluid Shear Stress Stimulates Incorporation of Hyaluronan Into Endothelial Cell Glycocalyx. Am. J. Physiol. Heart Circulatory Physiol. 290 (1), H458–H452. doi: 10.1152/ajpheart.00592.2005 16126814

[B73] Groomes P. V. Kanjee U. Duraisingh M. T. (2022). RBC Membrane Biomechanics and *Plasmodium Falciparum* Invasion: Probing Beyond Ligand-Receptor Interactions. Trends Parasitol. 38 (4), 301–315. doi: 10.1016/j.pt.2021.12.005 PMC8917059 34991983

[B74] Grüring C. Heiber A. Kruse F. Ungefehr J. Gilberger T.-W. Spielmann T. (2011). Development and Host Cell Modifications of *Plasmodium Falciparum* Blood Stages in Four Dimensions. Nat. Commun. 2 (1), 165. doi: 10.1038/ncomms1169 21266965

[B75] Handayani S. Chiu D. T. Tjitra E. Kuo J. S. Lampah D. Kenangalem E. (2009). High Deformability of *Plasmodium Vivax*-Infected Red Blood Cells Under Microfluidic Conditions. J. Infect. Dis. 199 (3), 445–450. doi: 10.1086/596048 19090777 PMC4337984

[B76] Hanssen E. Knoechel C. Klonis N. Abu-Bakar N. Deed S. LeGros M. (2011). Cryo Transmission X-Ray Imaging of the Malaria Parasite, *P. Falciparum* . J. Struct. Biol. 173 (1), 161–168. doi: 10.1016/j.jsb.2010.08.013 20826218 PMC3005799

[B77] Hanssen E. Dekiwadia C. Riglar D. T. Rug M. Lemgruber L. Cowman A. F. (2013). Electron Tomography of *Plasmodium Falciparum* Merozoites Reveals Core Cellular Events That Underpin Erythrocyte Invasion: Ultrastructure of the Invading Malaria Parasite. Cell. Microbiol. 15 (9), 1457–1472. doi: 10.1111/cmi.12132 23461734

[B78] Haymet A. B. Bartnikowski N. Wood E. S. Vallely M. P. McBride A. Yacoub S. (2021). Studying the Endothelial Glycocalyx *In Vitro*: What Is Missing? Front. Cardiovasc. Med. 8. doi: 10.3389/fcvm.2021.647086 PMC8079726 33937360

[B79] Helfrich W. Servuss R. M. (1984). Undulations, Steric Interaction and Cohesion of Fluid Membranes. Il. Nuovo Cimento D. 3 (1), 137–151. doi: 10.1007/bf02452208

[B80] Helms G. Dasanna A. K. Schwarz U. S. Lanzer M. (2016). Modeling Cytoadhesion of *Plasmodium Falciparum*-Infected Erythrocytes and Leukocytes-Common Principles and Distinctive Features. FEBS Lett. 590 (13), 1955–1971. doi: 10.1002/1873-3468.12142 26992823 PMC5071704

[B81] Hempel C. Pasini E. M. Kurtzhals J. A. L. (2016). Endothelial Glycocalyx: Shedding Light on Malaria Pathogenesis. Trends Mol. Med. 22 (6), 453–457. doi: 10.1016/j.molmed.2016.04.004 27161599

[B82] Herricks T. Antia M. Rathod P. K. (2019). Deformability Limits of *Plasmodium Falciparum*-Infected Red Blood Cells. Cell. Microbiol. 11 (9), 1340–1353. doi: 10.1111/j.1462-5822.2009.01334.x PMC2774476 19438513

[B83] Hillringhaus S. Dasanna A. K. Gompper G. Fedosov D. A. (2009). Importance of Erythrocyte Deformability for the Alignment of Malaria Parasite Upon Invasion. Biophys. J. 117 (7), 1202–1214. doi: 10.1016/j.bpj.2019.08.027 PMC6818165 31540708

[B84] Hillringhaus S. Dasanna A. K. Gompper G. Fedosov D. A. (2020). Stochastic Bond Dynamics Facilitates Alignment of Malaria Parasite at Erythrocyte Membrane Upon Invasion. ELife 9, e56500. doi: 10.7554/eLife.56500 32420874 PMC7269671

[B85] Hosseini S. M. Feng J. J. (2012). How Malaria Parasites Reduce the Deformability of Infected Red Blood Cells. Biophys. J. 103 (1), 1–10. doi: 10.1016/j.bpj.2012.05.026 22828326 PMC3388224

[B86] Introini V. Crick A. Tiffert T. Kotar J. Lin Y. C. Cicuta P. (2018a). Evidence Against a Role of Elevated Intracellular Ca2+ During *Plasmodium Falciparum* Preinvasion. Biophys. J. 114 (7), 1695–1706. doi: 10.1016/j.bpj.2018.02.023 29642038 PMC5954356

[B87] Introini V. Carciati A. Tomaiuolo G. Cicuta P. abd Guido S. (2018b). Endothelial Glycocalyx Regulates Cytoadherence in *Plasmodium Falciparum* Malaria. J. R. Soc. Interface 15 (149), 20180773. doi: 10.1098/rsif.2018.0773 30958233 PMC6303788

[B88] Introini V. Marin-Menendez A. Nettesheim G. Lin Y.-C. Kariuki S. N. Smith A. L. (2022). The Erythrocyte Membrane Properties of Beta Thalassaemia Heterozygotes and Their Consequences for *Plasmodium Falciparum* Invasion. Cold Spring Harbor Laboratory doi: 10.1101/2022.01.13.476158 PMC9142571 35624125

[B89] Ishida S. Ami A. Imai Y. (2017). Factors Diminishing Cytoadhesion of Red Blood Cells Infected by *Plasmodium Falciparum* in Arterioles. Biophys. J. 113 (5), 1163–1172. doi: 10.1016/j.bpj.2017.07.020 28877497 PMC5613969

[B90] Jaumouillé V. Cartagena-Rivera A. X. Waterman C. M. (2019). Coupling of β2 Integrins to Actin by a Mechanosensitive Molecular Clutch Drives Complement Receptor-Mediated Phagocytosis. Nat. Cell Biol. 21 (11), 1357–1369. doi: 10.1038/s41556-019-0414-2 31659275 PMC6858589

[B91] Kabaso D. Shlomovitz R. Auth T. Lew V. L. Gov N. S. (2010). Curling and Local Shape Changes of Red Blood Cell Membranes Driven by Cytoskeletal Reorganization. Biophys. J. 99 (3), 808–816. doi: 10.1016/j.bpj.2010.04.067 20682258 PMC2913190

[B92] Kariuki S. N. Marin-Menendez A. Introini V. Ravenhill B. J. Lin Y. C. Macharia A. (2020). Red Blood Cell Tension Protects Against Severe Malaria in the Dantu Blood Group. Nature 585, 579–583. doi: 10.1038/s41586-020-2726-6 32939086 PMC7116803

[B93] Kariuki S. N. Williams T. N. (2020). Human Genetics and Malaria Resistance. Hum. Genet. 139 (6-7), 801–811. doi: 10.1007/s00439-020-02142-6 32130487 PMC7271956

[B94] Kataoka H. Ushiyama A. Kawakami H. Akimoto Y. Matsubara S. Iijima T. (2015). Fluorescent Imaging of Endothelial Glycocalyx Layer With Wheat Germ Agglutinin Using Intravital Microscopy. Microscopy Res. Tech. 79 (1), 31–37. doi: 10.1002/jemt.22602 26768789

[B95] Keeling P. J. Rayner J. C. (2015). The Origins of Malaria: There Are More Things in Heaven and Earth. Parasitology 142, S16–S25. doi: 10.1017/S0031182014000766 24963725 PMC4413824

[B96] Koch M. Wright K. E. Otto O. Herbig M. Salinas N. D. Tolia N. H. (2017). *Plasmodium Falciparum* Erythrocyte-Binding Antigen 175 Triggers a Biophysical Change in the Red Blood Cell That Facilitates Invasion. PNAS 114 (16), 4225–4230. doi: 10.1073/pnas.1620843114 28373555 PMC5402469

[B97] Kolářová H. Ambrůzová B. Švihálková Šindlerová L. Klinke A. Kubala L. (2014). Modulation of Endothelial Glycocalyx Structure Under Inflammatory Conditions. Mediators Inflammation 2014, 694312. doi: 10.1155/2014/694312 PMC3997148 24803742

[B98] Koutsiaris A. G. Tachmitzi S. V. Batis N. (2013). Wall Shear Stress Quantification in the Human Conjunctival Pre-Capillary Arterioles *In Vivo* . Microvascular Res. 85, 34–39. doi: 10.1016/j.mvr.2012.11.003 23154279

[B99] Krishnan R. Klumpers D. D. Park C. Y. Rajendran K. Trepat X. van Bezu J. (2011). Substrate Stiffening Promotes Endothelial Monolayer Disruption Through Enhanced Physical Forces. Am. J. Physiol. Cell Physiol. 300 (1), C146–C154. doi: 10.1152/ajpcell.00195.2010 20861463 PMC3023192

[B100] Kumar K. Srinivasan P. Nold M. J. Moch J. K. Reiter K. Sturdevant D. (2017). Profiling Invasive *Plasmodium Falciparum* Merozoites Using an Integrated Omics Approach. Sci. Rep. 7 (1), 17146. doi: 10.1038/s41598-017-17505-9 29215067 PMC5719419

[B101] Lanotte L. Tomaiuolo G. Misbah C. Bureau L. Guido S. (2014). Red Blood Cell Dynamics in Polymer Brush-Coated Microcapillaries: A Model of Endothelial Glycocalyx. Vitro Biomicrofluidics 8 (1), 014104. doi: 10.1063/1.4863723 24753725 PMC3977877

[B102] Lansche C. Dasanna A. K. Quadt K. Fröhlich B. Missirlis D. Tétard M. (2018). The Sickle Cell Trait Affects Contact Dynamics and Endothelial Cell Activation in *Plasmodium Falciparum*-Infected Erythrocytes. Commun. Biol. 1 (1), 211. doi: 10.1038/s42003-018-0223-3 30534603 PMC6269544

[B103] Lavazec C. (2017). Molecular Mechanisms of Deformability of P*lasmodium*-Infected Erythrocytes. Curr. Opin. Microbiol. 40, 138–144. doi: 10.1016/j.mib.2017.11.011 29175339

[B104] Lee M. C. S. Lindner S. E. Lopez-Rubio J. J. Llinás M. (2019). Cutting Back Malaria: CRISPR/Cas9 Genome Editing of *Plasmodium. Briefings in Functional* . Genomics 18 (5), 281–289. doi: 10.1093/bfgp/elz012 PMC6859820 31365053

[B105] Lee W.-C. Shahari S. Nguee S. Y.T. Lau Y. L. Rénia L. (2021). Cytoadherence Properties of *Plasmodium knowlesi*-infected erythrocytes. Front. Microbiol. 12. doi: 10.3389/fmicb.2021.804417 PMC8767020 35069511

[B106] Lee W.-C. Malleret B. Lau Y. L. Mauduit M. Fong M. Y. Cho J. S.. (2014). Glycophorin C (CD236R) Mediates *Vivax* Malaria Parasite Rosetting to Normocytes. Blood 123 (18), e100–e109. doi: 10.1182/blood-2013-12-541698 24652986 PMC4007619

[B107] Leffler E. M. Band G. Busby G. B.J. Kivinen K. Le Q. S. Clarke G. M. (2017). Resistance to Malaria Through Structural Variation of Red Blood Cell Invasion Receptors. Science 356 (6343). doi: 10.1126/science.aam6393 PMC5575826 28522690

[B108] Lennartz F. Adams Y. Bengtsson A. Olsen R. W. Turner L. Ndam N. T. (2017). Structure-Guided Identification of a Family of Dual Receptor-Binding *Pf*EMP1 That is Associated With Cerebral Malaria. Cell Host Microbe 21 (3), 403–414. doi: 10.1016/j.chom.2017.02.009 28279348 PMC5374107

[B109] Lessard S. Gatof E. S. Beaudoin M. Schupp P. G. Sher F. Ali A. (2017). An Erythroid-Specific ATP2B4 Enhancer Mediates Red Blood Cell Hydration and Malaria Susceptibility. J. Clin. Invest. 127 (8), 3065–3074. doi: 10.1172/JCI94378 28714864 PMC5531409

[B110] Lew V. L. (2005). Malaria: Endless Fascination With Merozoite Release. Curr. Biol. 15 (18), R760–R761. doi: 10.1016/j.cub.2005.08.059 16169475

[B111] Lew V. L. (2011). Malaria: Surprising Mechanism of Merozoite Egress Revealed. Curr. Biol. 21 (9), R314–R316. doi: 10.1016/j.cub.2011.03.066 21549952

[B112] Lew V. L. Hockaday A. Freeman C. J. Bookchin R. M. (1988). Mechanism of Spontaneous Inside-Out Vesiculation of Red Cell Membranes. J. Cell Biol. 106 (6), 1893–1901. doi: 10.1083/jcb.106.6.1893 3384849 PMC2115135

[B113] Lew V. L. Tiffert T. (2007). Is Invasion Efficiency in Malaria Controlled by Pre-Invasion Events? Trends Parasitol. 23 (10), 481–484. doi: 10.1016/j.pt.2007.08.001 17804296

[B114] Lew V. L. Tiffert T. (2017). On the Mechanism of Human Red Blood Cell Longevity: Roles of Calcium, the Sodium Pump, PIEZO1, and Gardos Channels. Front. Physiol. 8. doi: 10.3389/fphys.2017.00977 PMC5732905 29311949

[B115] Lim H. W. G. Wortis M. Mukhopadhyay R. (2002). Stomatocyte-Discocyte-Echinocyte Sequence of the Human Red Blood Cell: Evidence for the Bilayer- Couple Hypothesis From Membrane Mechanics. PNAS 99 (26), 16766–16769. doi: 10.1073/pnas.202617299 12471152 PMC139218

[B116] Lim Y. B. Thingna J. Cao J. Lim C. T. (2017). Single Molecule and Multiple Bond Characterization of Catch Bond Associated Cytoadhesion in Malaria. Sci. Rep. 7 (1), 4208. doi: 10.1038/s41598-017-04352-x 28646215 PMC5482833

[B117] Liu W. Wang X. Bai K. Lin M. Sukhorukov G. Wang W. (2014). Microcapsules Functionalized With Neuraminidase can Enter Vascular Endothelial Cells *In Vitro* . J. R. Soc. Interface 11 (101), 20141027. doi: 10.1098/rsif.2014.1027 25339691 PMC4223926

[B118] Liu Y. Zhang T. Zhang H. Li J. Zhou N. Fiskesund R. (2021). Cell Softness Prevents Cytolytic T-Cell Killing of Tumor-Repopulating Cells. Cancer Res. 81 (2), 476–488. doi: 10.1158/0008-5472.CAN-20-2569 33168645

[B119] Long R. K. M. Piatti L. Korbmacher F. Bernabeu M. (2021). Understanding Parasite-Brain Microvascular Interactions With Engineered 3D Blood-Brain Barrier Models. Mol. Microbiol. 117 (3), 693–704. doi: 10.1111/mmi.14852 34837419

[B120] Lykov K. Li X. Lei H. Pivkin I. V. Karniadakis G. E. (2015). Inflow/Outflow Boundary Conditions for Particle-Based Blood Flow Simulations: Application to Arterial Bifurcations and Trees. PloS Comput. Biol. 11 (8), e1004410. doi: 10.1371/journal.pcbi.1004410 26317829 PMC4552763

[B121] Ma S. Cahalan S. LaMonte G. Grubaugh N. D. Zeng W. Murthy S. E. (2018). Common PIEZO1 Allele in African Populations Causes RBC Dehydration and Attenuates *Plasmodium* Infection. Cell 173 (2), 443–455.e12. doi: 10.1016/j.cell.2018.02.047 29576450 PMC5889333

[B122] Mahmoud M. Cancel L. Tarbell J. M. (2021). Matrix Stiffness Affects Glycocalyx Expression in Cultured Endothelial Cells. Front. Cell Dev. Biol. 9. doi: 10.3389/fcell.2021.731666 PMC8530223 34692689

[B123] Malaria Genomic Epidemiology Network (2015). A Novel Locus of Resistance to Severe Malaria in a Region of Ancient Balancing Selection. Nature 526 (7572), 253–257. doi: 10.1038/nature15390 26416757 PMC4629224

[B124] Malleret B. Li A. Zhang R. Tan K. S. W. Suwanarusk R. Claser C. (2015). *Plasmodium Vivax*: Restricted Tropism and Rapid Remodeling of CD71-Positive Reticulocytes. Blood 125 (8), 1314–1324. doi: 10.1182/blood-2014-08-596015 25414440 PMC4401350

[B125] Marsh G. Waugh R. E. (2013). Quantifying the Mechanical Properties of the Endothelial Glycocalyx With Atomic Force Microscopy. J. Visualized Experiments 72, e50163. doi: 10.3791/50163 PMC3605807 23462566

[B126] Mauritz J. M. A. Esposito A. Ginsburg H. Kaminski C. F. Tiffert T. Lew V. L. (2009). The Homeostasis of *Plasmodium Falciparum*-Infected Red Blood Cells. PloS Comput. Biol. 5 (4), e1000339. doi: 10.1371/journal.pcbi.1000339 19343220 PMC2659444

[B127] Mauritz J. M. A. Esposito A. Tiffert T. Skepper J. N. Warley A. Yoon Y. Z. (2010). Biophotonic Techniques for the Study of Malaria-Infected Red Blood Cells. Med. Biol. Eng. Computing 48 (10), 1055–1063. doi: 10.1007/s11517-010-0668-0 20661776

[B128] Menard R. Tavares J. Cockburn I. Markus M. Zavala F. Amino R. (2013). Looking Under the Skin: The First Steps in Malarial Infection and Immunity. Nat. Rev. Microbiol. 11, 701–712. doi: 10.1038/nrmicro3111 24037451

[B129] Mikkelsen R. B. Tanabe K. Wallach D. F. (1982). Membrane Potential of *Plasmodium-*Infected Erythrocytes. J. Cell Biol. 93 (3), 685–689. doi: 10.1083/jcb.93.3.685 6288730 PMC2112149

[B130] Mikkelsen R. B. Wallach D. F. H. Van Doren E. Nillni E. A. (1986). Membrane Potential of Erythrocytic Stages of *Plasmodium Chabaudi* Free of the Host Cell Membrane. Mol. Biochem. Parasitol. 21 (1), 83–92. doi: 10.1016/0166-6851(86)90082-4 3773936

[B131] Miller L. H. Aikawa M. Johnson J. G. Shiroishi T. (1979). Interaction Between Cytochalasin B-Treated Malarial Parasites and Erythrocytes. Attachment and Junction Formation. J. Exp. Med. 149 (1), 172–184. doi: 10.1084/jem.149.1.172 105074 PMC2184746

[B132] Mills J. P. Diez-Silva M. Quinn D. J. Dao M. Lang M. J. Tan K. S. W. (2007). Effect of Plasmodial RESA Protein on Deformability of Human Red Blood Cells Harboring. PNAS 104 (22), 9213–9217. doi: 10.1073/pnas.0703433104 17517609 PMC1874230

[B133] Milner D. A. Lee J. J. Frantzreb C. Whitten R. O. Kamiza S. Carr R. A. (2015). Quantitative Assessment of Multiorgan Sequestration of Parasites in Fatal Pediatric Cerebral Malaria. J. Infect. Dis. 212 (8), 1317–1321. doi: 10.1093/infdis/jiv205 25852120 PMC4577044

[B134] Mognetti B. M. Cicuta P. Di Michele L. (2019). Programmable Interactions With Biomimetic DNA Linkers at Fluid Membranes and Interfaces. Rep. Prog. Phys. 82 (11), 116601. doi: 10.1088/1361-6633/ab37ca 31370052

[B135] Mohandas N. Lie-Injo L. Friedman M. Mak J. (1984). Rigid Membranes of Malayan Ovalocytes: A Likely Genetic Barrier Against Malaria. Blood 63 (6), 1385–1392. doi: 10.1182/blood.V63.6.1385.1385 6722355

[B136] Monzel C. Sengupta K. (2016). Measuring Shape Fluctuations in Biological Membranes. J. Phys. D.: Appl. Phys. 49 (24), 243002. doi: 10.1088/0022-3727/49/24/243002

[B137] Moore K. H. Murphy H. A. George E. M. (2021). The Glycocalyx: A Central Regulator of Vascular Function. Am. J. Physiol. Regulatory Integr. Comp. Physiol. 320 (4), R508–R518. doi: 10.1152/ajpregu.00340.2020 PMC8238147 33501896

[B138] Mota M. M. Pradel G. Vanderberg J. P. Hafalla J. C. R. Frevert U. Nussenzweig R. S. (2001). Migration of *Plasmodium* Sporozoites Through Cells Before Infection. Science 291, 141–144. doi: 10.1126/science.291.5501.141 11141568

[B139] Namvar A. Blanch A. J. Dixon M. W. Carmo O. M. S. Liu B. Tiash S. (2021). Surface Area-to-Volume Ratio, Not Cellular Viscoelasticity, Is the Major Determinant of Red Blood Cell Traversal Through Small Channels. Cell. Microbiol. 23 (1), e13270. doi: 10.1111/cmi.13270 32981231 PMC7757199

[B140] Narla J. Mohandas N. (2017). Red Cell Membrane Disorders. Int. J. Lab. Hematol. 39 (S1), 47–52. doi: 10.1111/ijlh.12657 28447420

[B141] Nash G. B. O’Brien E. Gordon-Smith E. Dormandy J. (1989). Abnormalities in the Mechanical Properties of Red Blood Cells Caused by *Plasmodium falciparum* . Blood 1 74 (2), 855–861. doi: 10.1182/blood.V74.2.855.bloodjournal742855 2665857

[B142] Ndila C. M. Uyoga S. Macharia A. W. Nyutu G. Peshu N. Ojal J. (2018). Human Candidate Gene Polymorphisms and Risk of Severe Malaria in Children in Kilifi, Kenya: A Case-Control Association Study. Lancet Haematol. 5 (8), e333–e345. doi: 10.1016/S2352-3026(18)30107-8 30033078 PMC6069675

[B143] Ndour P. A. Larréché S. Mouri O. Argy N. Gay F. Roussel C. (2017). Measuring the *Plasmodium Falciparum* HRP2 Protein in Blood From Artesunate-Treated Malaria Patients Predicts Post-Artesunate Delayed Hemolysis. Sci. Trans. Med. 9 (397), eaaf9377. doi: 10.1126/scitranslmed.aaf9377 28679662

[B144] Nguetse C. N. Purington N. Ebel E. R. Shakya B. Tetard M. Kremsner P. G. (2020). A Common Polymorphism in the Mechanosensitive Ion Channel PIEZO1 Is Associated With Protection From Severe Malaria in Humans. PNAS 117 (16), 9074–9081. doi: 10.1073/pnas.1919843117 32265284 PMC7183233

[B145] Niko Y. Didier P. Mely Y. Konishi G. Klymchenko A. S. (2016). Bright and Photostable Push-Pull Pyrene Dye Visualizes Lipid Order Variation Between Plasma and Intracellular Membranes. Sci. Rep. 6 (1), 18870. doi: 10.1038/srep18870 26750324 PMC4707542

[B146] Nyarko P. B. Tarr S. J. Aniweh Y. Stewart L. B. Conway D. J. Awandare G. A. (2020). Investigating a *Plasmodium Falciparum* Erythrocyte Invasion Phenotype Switch at the Whole Transcriptome Level. Sci. Rep. 10, 245. doi: 10.1038/s41598-019-56386-y 31937828 PMC6959351

[B147] Ockenhouse C. F. Betageri R. Springer T. A. Staunton D. E. (1992). *Plasmodium Falciparum*-Infected Erythrocytes Bind ICAM-1 at a Site Distinct From LFA-1, Mac-1, and Human Rhinovirus. Cell 68 (1), 63–69. doi: 10.1016/0092-8674(92)90206-r 1346257

[B148] Pais T. F. Penha-Gonçalves C. (2018). Brain Endothelium: The “Innate Immunity Response Hypothesis” in Cerebral Malaria Pathogenesis. Front. Immunol. 9. doi: 10.3389/fimmu.2018.03100 PMC6361776 30761156

[B149] Park Y. Diez-Silva M. Popescu G. Lykotrafitis G. Choi W. Feld M. S. (2008). Refractive Index Maps and Membrane Dynamics of Human Red Blood Cells Parasitized by *Plasmodium Falciparum* . PNAS 105 (37), 13730–13735. doi: 10.1073/pnas.0806100105 18772382 PMC2529332

[B150] Park Y. Best C. A. Badizadegan K. Dasari R. R. Feld M. S. Kuriabova T. (2010). Measurement of Red Blood Cell Mechanics During Morphological Changes. PNAS 107 (15), 6731–6736. doi: 10.1073/pnas.0909533107 20351261 PMC2872375

[B151] Paul D. Achouri S. Yoon Y. Z. Herre J. Bryant C. E. Cicuta P. (2013). Phagocytosis Dynamics Depends on Target Shape. Biophys. J. 105 (5), 1143–1150. doi: 10.1016/j.bpj.2013.07.036 24010657 PMC3762343

[B152] Paul A. Ramdani G. Tatu U. Langsley G. Natarajan V. (2019). Studying the Rigidity of Red Blood Cells Induced by *Plasmodium Falciparum* Infection. Sci. Rep. 9 (1), 6336. doi: 10.1038/s41598-019-42721-w 31004094 PMC6474899

[B153] Pavlou G. Biesaga M. Touquet B. Lagal V. Balland M. Dufour A. (2018). *Toxoplasma* Parasite Twisting Motion Mechanically Induces Host Cell Membrane Fission to Complete Invasion Within a Protective Vacuole. Cell Host Microbe 24 (1), 81–96.e5. doi: 10.1016/j.chom.2018.06.003 30008293

[B154] Popescu G. Ikeda T. Goda K. Best-Popescu C. A. Laposata M. Manley S. (2006). Optical Measurement of Cell Membrane Tension. Phys. Rev. Lett. 97 (21), 218101. doi: 10.1103/PhysRevLett.97.218101 17155774

[B155] Pries A. R. Secomb T. W. (2005). Microvascular Blood Viscosity *In Vivo* and the Endothelial Surface Layer. Am. J. Physiol. Heart Circulatory Physiol. 289 (6), H2657–H2664. doi: 10.1152/ajpheart.00297.2005 16040719

[B156] Prudencio M. Rodriguez A. Mota M. M. (2006).The Silent Path to Thousands of Merozoites: The P*lasmodium* Liver Stage. Nat. Rev. Microbiol. 4, 849–856, 2006. doi: 10.1038/nrmicro1529 17041632

[B157] Raasch M. Rennert K. Jahn T. Peters S. Henkel T. Huber O. (2015). Microfluidically Supported Biochip Design for Culture of Endothelial Cell Layers With Improved Perfusion Conditions. Biofabrication 7 (1), 15013. doi: 10.1088/1758-5090/7/1/015013 25727374

[B158] Rayner S. G. Howard C. C. Mandrycky C. J. Stamenkovic S. Himmelfarb J. (2021). Multiphoton-Guided Creation of Complex Organ-Specific Microvasculature. Adv. Healthc. Mater. 10 (10), e2100031. doi: 10.1002/adhm.202100031 33586357 PMC8137585

[B159] Reid H. L. Barnes A. J. Lock P. J. Dormandy J. A. Dormandy T. L. (1976). A Simple Method for Measuring Erythrocyte Deformability. J. Clin. Pathol. 29 (9), 855–858. doi: 10.1136/jcp.29.9.855 977787 PMC476193

[B160] Reinitz A. DeStefano J. Ye M. Wong A. D. Searson P. C. (2015). Human Brain Microvascular Endothelial Cells Resist Elongation Due to Shear Stress. Microvascular Res. 99, 8–18. doi: 10.1016/j.mvr.2015.02.008 PMC4426013 25725258

[B161] Rigat-Brugarolas L. G. Elizalde-Torrent A. Bernabeu M. De Niz M. Martin-Jaular L. Fernandez-Becerra C.. (2014). A Functional Microengineered Model of the Human Spleenon-on-a-Chip. Lab. Chip. 14 (10), 1715–1724. doi: 10.1039/c3lc51449h 24663955

[B162] Riggle B. A. Manglani M. Maric D. Johnson K. R. Lee M.-H. Neto O. L.A. (2020). CD8+ T Cells Target Cerebrovasculature in Children With Cerebral Malaria. J. Clin. Invest. 130 (3), 1128–1138. doi: 10.1172/JCI133474 31821175 PMC7269583

[B163] Riglar D. T. Richard D. Wilson D. W. Boyle M. J. Dekiwadia C. Turnbull L. (2011). Super-Resolution Dissection of Coordinated Events During Malaria Parasite Invasion of the Human Erythrocyte. Cell Host Microbe 9 (1), 9–20. doi: 10.1016/j.chom.2010.12.003 21238943

[B164] Rogers S. Lew V. L. (2021). Up-Down Biphasic Volume Response of Human Red Blood Cells to PIEZO1 Activation During Capillary Transits. PloS Comput. Biol. 17 (3), e1008706. doi: 10.1371/journal.pcbi.1008706 33657092 PMC7928492

[B165] Rudlaff R. M. Kraemer S. Marshman J. Dvorin J. D. (2020). Three-Dimensional Ultrastructure of *Plasmodium Falciparum* Throughout Cytokinesis. PloS Pathog. 16 (6), e1008587. doi: 10.1371/journal.ppat.1008587 32511279 PMC7302870

[B166] Rug M. Prescott S. W. Fernandez K. M. Cooke B. M. Cowman A. F. (2006). The Role of KAHRP Domains in Knob Formation and Cytoadherence of *P. Falciparum*-Infected Human Erythrocytes. Blood 108 (1), 370–378. doi: 10.1182/blood-2005-11-4624 16507777 PMC1895844

[B167] Russell B. M. Cooke B. M. (2017). The Rheopathobiology of *Plasmodium vivax* and Other Important Primate Malaria Parasites. Trends Parasitol. 33 (4), 321–334. doi: 10.1016/j.pt.2016.11.009 28040374

[B168] Safeukui I. Buffet P. A. Perrot S. Sauvanet A. Aussilhou B. Dokmak S. (2013). Surface Area Loss and Increased Sphericity Account for the Splenic Entrapment of Subpopulations of *Plasmodium Falciparum* Ring-Infected Erythrocytes. PloS One 8, e60150. doi: 10.1371/journal.pone.0060150 23555907 PMC3610737

[B169] Saint-Sardos A. Sart S. Lippera K. Brient‐Litzler E. Michelin S. Amselem G. (2020). High-Throughput Measurements of Intra-Cellular and Secreted Cytokine From Single Spheroids Using Anchored Microfluidic Droplets. Small 16 (49), e2002303. doi: 10.1002/smll.202002303 33185938

[B170] Sanchez C. P. Patra P. Chang S. S. Karathanasis C. Hanebutte L. Kilian N.. (2022). KAHRP Dynamically Relocalizes to Remodeled Actin Junctions and Associates With Knob Spirals in *Plasmodium Falciparum*-Infected Erythrocytes. Mol. Microbiol. 117 (2), 274–292. doi: 10.1111/mmi.14811 34514656

[B171] Sanyal S. Egée S. Bouyer G. Perrot S. Safeukui I. Bischoff E. (2012). *Plasmodium Falciparum* STEVOR Proteins Impact Erythrocyte Mechanical Properties. Blood 119 (2), e1–e8. doi: 10.1182/blood-2011-08-370734 22106347 PMC3257022

[B172] Schrier S. L. Rachmilewitz E. Mohandas N. (1989). Cellular and Membrane Properties of Alpha and Beta Thalassemic Erythrocytes are Different: Implication for Differences in Clinical Manifestations. Blood 74 (6), 2194–2202. doi: 10.1182/blood.v74.6.2194.2194 2804358

[B173] Scott H. A. Quach B. Yang X. Ardekani S. Cabrera A. P. Wilson R. (2016). Matrix Stiffness Exerts Biphasic Control Over Monocyte–Endothelial Adhesion *via* Rho-Mediated ICAM-1 Clustering. Integr. Biol.: Quantitative Biosci. Nano Macro 8 (8), 869–878. doi: 10.1039/c6ib00084c 27444067

[B174] Seydel K. B. Kampondeni S. D. Valim C. Potchen M. J. Milner D. A. Muwalo F. W. (2015). Brain Swelling and Death in Children With Cerebral Malaria. New Engl. J. Med. 372 (12), 1126–1137. doi: 10.1056/NEJMoa1400116 25785970 PMC4450675

[B175] Shrirao A. B. Schloss R. S. Fritz Z. Shrirao M. V. Rosen R. Yarmush M. L. (2021). Autofluorescence of Blood and Its Application in Biomedical and Clinical Research. Biotechnol. Bioengineer. 118 (12), 4550–4576. doi: 10.1002/bit.27933 34487351

[B176] Sinha A. Chu T. T.T. Dao M. Chandramohanadas R. (2015). Single-Cell Evaluation of Red Blood Cell Bio-Mechanical and Nano-Structural Alterations Upon Chemically Induced Oxidative Stress. Sci. Rep. 5, 9768. doi: 10.1038/srep09768 25950144 PMC4423428

[B177] Sinha R. Le Gac S. Verdonschot N. van denBerg A. Koopman B. Rouwkema J. (2016). Endothelial Cell Alignment as a Result of Anisotropic Strain and Flow Induced Shear Stress Combinations. Sci. Rep. 6 (1), 29510. doi: 10.1038/srep29510 27404382 PMC4941569

[B178] Sisquella X. Nebl T. Thompson J. K. Whitehead L. Malpede B. M. Salinas N. D. (2017). *Plasmodium Falciparum* Ligand Binding to Erythrocytes Induce Alterations in Deformability Essential for Invasion. ELife 6, 1–20. doi: 10.7554/eLife.21083 PMC5333951 28226242

[B179] Smith J. D. Rowe J. A. Higgins M. K. Lavstsen T. (2013). Malaria’s Deadly Grip: Cytoadhesion of *Plasmodium Falciparum*-Infected Erythrocytes. Cell. Microbiol. 15 (12), 1976–1983. doi: 10.1111/cmi.12183 23957661 PMC3836831

[B180] Storm J. Jespersen J. S. Seydel K. B. Szestak T. Mbewe M. Chisala N. V. (2019). Cerebral Malaria Is Associated With Differential Cytoadherence to Brain Endothelial Cells. EMBO Mol. Med. 11 (2), e9164. doi: 10.15252/emmm.201809164 30610112 PMC6365927

[B181] Suresh S. (2006). Mechanical Response of Human Red Blood Cells in Health and Disease: Some Structure-Property-Function Relationships. J. Mater. Res. 21, 1871–1877. doi: 10.1557/jmr.2006.0260

[B182] Suresh S. Spatz J. Mills J. P. Micoulet A. Dao M. Lim C. T. (2005). Connections Between Single-Cell Biomechanics and Human Disease States: Gastrointestinal Cancer and Malaria. Acta Biomater. 1, 15–30. doi: 10.1016/j.actbio.2004.09.001 16701777

[B183] Suwanarusk R. Cooke B. M. Dondorp A. M. Silamut K. Sattabongkot J. White N. J. (2004). The Deformability of Red Blood Cells Parasitized by Plasmodium falciparum and P. *vivax* . J. Infect. Dis. 189, 2, 190–194. doi: 10.1086/380468 14722882

[B184] Tan S. Y. Leung Z. Wu A. R. (2020). Recreating Physiological Environments *In Vitro*: Design Rules for Microfluidic-Based Vascularized Tissue Constructs. Small 16 (9), e1905055. doi: 10.1002/smll.201905055 31913580

[B185] Tapia J. Vera N. Aguilar J. González M. Sánchez S. A. Coelho P. (2021). Correlated Flickering of Erythrocytes Membrane Observed With Dual Time Resolved Membrane Fluctuation Spectroscopy Under Different D-Glucose Concentrations. Sci. Rep. 11 (1), 2429. doi: 10.1038/s41598-021-82018-5 33510337 PMC7844050

[B186] Tavares J. Formaglio P. Thiberge S. Mordelet E. Van Rooijen N. Medvinsky A. (2013). Role of Host Cell Traversal by the Malaria Sporozoite During Liver Infection. J. Exp. Med. 210, 905–991. doi: 10.1084/jem.20121130 23610126 PMC3646492

[B187] Tello-Lafoz M. Srpan K. Sanchez E. E. Hu J. Remsik J. Romin Y. (2021). Cytotoxic Lymphocytes Target Characteristic Biophysical Vulnerabilities in Cancer. Immunity 54 (5), 1037–1054.e7. doi: 10.1016/j.immuni.2021.02.020 33756102 PMC8119359

[B188] Tham W.-H. Lim N. T.Y. Weiss G. E. Lopaticki S. Ansell B. R.E. Bird M. (2015). *Plasmodium Falciparum* Adhesins Play an Essential Role in Signalling and Activation of Invasion Into Human Erythrocytes. PloS Pathog. 11 (12), e1005343. doi: 10.1371/journal.ppat.1005343 26694741 PMC4687929

[B189] Thye T. Evans J. A. Ruge G. Loag W. Ansong D. Agbenyega T. (2022). Human Genetic Variant E756del in the Ion Channel PIEZO1 Not Associated With Protection From Severe Malaria in a Large Ghanaian Study. J. Hum. Genet. 67 (1), 65–67. doi: 10.1038/s10038-021-00958-2 34230590 PMC8727285

[B190] Tiffert T. Lew V. L. Ginsburg H. Krugliak M. Croisille L. Mohandas N. (2005). The Hydration State of Human Red Blood Cells and Their Susceptibility to Invasion by *Plasmodium Falciparum* . Blood 105 (12), 4853–4860. doi: 10.1182/blood-2004-12-4948 15728121 PMC1894996

[B191] Tiffert T. Lew V. L. (2014). Dynamic Morphology and Cytoskeletal Protein Changes During Spontaneous Inside-Out Vesiculation of Red Blood Cell Membranes. Pflugers Archiv.: Eur. J. Physiol. 466 (12), 2279–2288. doi: 10.1007/s00424-014-1483-5 24615169 PMC4233320

[B192] Timmann C. Thye T. Vens M. Evans J. May J. Ehmen C. (2012). Genome-Wide Association Study Indicates Two Novel Resistance Loci for Severe Malaria. Nature 489 (7416), 443–446. doi: 10.1038/nature11334 22895189

[B193] Totino P. R. Lopes S. C. (2017). Insights Into the Cytoadherence Phenomenon of *Plasmodium Vivax*: The Putative Role of Phosphatidylserine. Front. Immunol. 8. doi: 10.3389/fimmu.2017.01148 PMC5611623 28979260

[B194] Treeck M. Zacherl S. Herrmann S. Cabrera A. Kono M. Struck N. S. (2009). Functional Analysis of the Leading Malaria Vaccine Candidate AMA-1 Reveals an Essential Role for the Cytoplasmic Domain in the Invasion Process. PloS Pathog. 5 (3), e1000322. doi: 10.1371/journal.ppat.1000322 19283086 PMC2654807

[B195] Tsvirkun D. Grichine A. Duperray A. Misbah C. Bureau L. (2017). Microvasculature on a Chip: Study of the Endothelial Surface Layer and the Flow Structure of Red Blood Cells. Sci. Rep. 7, 45036. doi: 10.1038/srep45036 28338083 PMC5364477

[B196] Turner L. Lavstsen T. Berger S. S. Wang C. W. Petersen J. E.V. Avril M. (2013). Severe Malaria Is Associated With Parasite Binding to Endothelial Protein C Receptor. Nature 498 (7455), 502–505. doi: 10.1038/nature12216 23739325 PMC3870021

[B197] Udomsangpetch R. Pipitaporn B. Silamut K. Pinches R. Kyes S. Looareesuwan S. (2002). Febrile Temperatures Induce Cytoadherence of Ring-Stage *Plasmodium Falciparum*-Infected Erythrocytes. PNAS 99, 11825–11829. doi: 10.1073/pnas.172398999 12177447 PMC129353

[B198] Valanciunaite J. Kempf E. Seki H. Danylchuk D. I. Peyriéras N. Niko Y. Klymchenko A.. (2020). Polarity Mapping of Cells and Embryos by Improved Fluorescent Solvatochromic Pyrene Probe. Analytical Chem. 92 (9), 6512–6520. doi: 10.1021/acs.analchem.0c00023 32153188

[B199] Velasco S. Kedaigle A. J. Simmons S. K. Nash A. Rocha M. Quadrato G. (2019). Individual Brain Organoids Reproducibly Form Cell Diversity of the Human Cerebral Cortex. Nature 570 (7762), 523–527. doi: 10.1038/s41586-019-1289-x 31168097 PMC6906116

[B200] Villegas-Mendez A. Stafford N. Haley M. J. Pravitasari N. E. Baudoin F. Ali A. (2021). The Plasma Membrane Calcium ATPase 4 Does Not Influence Parasite Levels But Partially Promotes Experimental Cerebral Malaria During Murine Blood Stage Malaria. Malaria J. 20 (1), 297. doi: 10.1186/s12936-021-03832-w PMC8252299 34215257

[B201] Vorselen D. Barger S. R. Wang Y. Cai W. Theriot J. A. Gauthier N. C. (2021). Phagocytic “Teeth” and Myosin-II “Jaw” Power Target Constriction During Phagocytosis. ELife 10, e68627. doi: 10.7554/eLife.68627 34708690 PMC8585483

[B202] Waldecker M. Dasanna A. K. Lansche C. Linke M. Srismith S. Cyrklaff M. (2017). Differential Time-Dependent Volumetric and Surface Area Changes and Delayed Induction of New Permeation Pathways in *P. Falciparum*-Infected Hemoglobinopathic Erythrocytes. Cell. Microbiol. 19 (2), e12650. doi: 10.1111/cmi.12650 PMC5298026 27450804

[B203] Wang W. Lollis E. M. Bordeleau F. Reinhart-King C. A. (2019). Matrix Stiffness Regulates Vascular Integrity Through Focal Adhesion Kinase Activity. FASEB 33 (1), 1199–1208. doi: 10.1096/fj.201800841R PMC6355084 30102569

[B204] Wei W. Cheng W. Dai W. Lu F. Cheng Y. Jiang T. (2022). A Nanodrug Coated With Membrane From Brain Microvascular Endothelial Cells Protects Against Experimental Cerebral Malaria. Nano Lett. 22 (1), 211–219. doi: 10.1021/acs.nanolett.1c03514 34967631

[B205] Weiss G. E. Gilson P. R. Taechalertpaisarn T. Tham W.-H. de Jong N. W.M. Harvey K. L. (2015). Revealing the Sequence and Resulting Cellular Morphology of Receptor-Ligand Interactions During *Plasmodium Falciparum* Invasion of Erythrocytes. PloS Pathog. 11 (2), e1004670. doi: 10.1371/journal.ppat.1004670 25723550 PMC4344246

[B206] Weiss G. E. Crabb B. S. Gilson P. R.. (2016). Overlaying Molecular and Temporal Aspects of Malaria Parasite Invasion. Trends Parasitol. 32 (4), 284–295. doi: 10.1016/j.pt.2015.12.007 26778295

[B207] Wetzel D. M. Schmidt J. Kuhlenschmidt M. S. Dubey J. P. Sibley L. D. (2005). Gliding Motility Leads to Active Cellular Invasion by *Cryptosporidium Parvum* Sporozoites. Infect. Immun. 73 (9), 5379–5387. doi: 10.1128/IAI.73.9.5379-5387.2005 16113253 PMC1231075

[B208] Wissing F. Sanchez C. P. Rohrbach P. Ricken S. Lanzer M. (2002). Illumination of the Malaria Parasite *Plasmodium Falciparum* Alters Intracellular Ph: Implications for Live Cell Imaging. J. Biol. Chem. 277 (40), 37747–37755. doi: 10.1074/jbc.m204845200 12140286

[B209] World Malaria Report (2021). Available at: https://www.who.int/teams/globalmalaria-programme/reports/world-malaria-report-2021

[B210] Xavier M. Rosendahl P. Herbig M. Kräter M. Spencer D. Bornhäuser M. (2016). Mechanical Phenotyping of Primary Human Skeletal Stem Cells in Heterogeneous Populations by Real-Time Deformability Cytometry. Integr. Biol.: Quantitative Biosci. Nano Macro 8 (5), 616–623. doi: 10.1039/c5ib00304k 26980074

[B211] Yahata K. Hart M. N. Davies H. Asada M. Wassmer S. C. Templeton T. J. (2021). Gliding Motility of *Plasmodium* Merozoites. PNAS 118 (48), e2114442118. doi: 10.1073/pnas.2114442118 34819379 PMC8651183

[B212] Yeo T. W. Weinberg J. B. Lampah D. A. Kenangalem E. Bush P. Chen Y. (2019). Glycocalyx Breakdown is Associated With Severe Disease and Fatal Outcome in *Plasmodium Falcipa*rum Malaria. Clin. Infect. Dis.: Off. Publ. Infect. Dis. Soc. America 69 (10), 1712–1720. doi: 10.1093/cid/ciz038 PMC6821254 30753363

[B213] Yoon Y.-Z. Kotar J. Yoon G. Cicuta P. (2008). The Nonlinear Mechanical Response of the Red Blood Cell. Phys. Biol. 5 (3), 36007. doi: 10.1088/1478-3975/5/3/036007 18698116

[B214] Yoon Y.-Z. Hong H. Brown A. Kim D. C. Kang D. J. Lew V. L. (2009). Flickering Analysis of Erythrocyte Mechanical Properties: Dependence on Oxygenation Level, Cell Shape, and Hydration Level. Biophys. J. 97 (6), 1606–1615. doi: 10.1016/j.bpj.2009.06.028 19751665 PMC2741588

[B215] Zhang Y. Huang C. Kim S. Golkaram M. Dixon M. W.A. Tilley L. (2015). Multiple Stiffening Effects of Nanoscale Knobs on Human Red Blood Cells Infected With *Plasmodium Falciparum* Malaria Parasite. PNAS 112 (19), 6068–6073. doi: 10.1073/pnas.1505584112 25918423 PMC4434686

[B216] Zhang R. Chandramohanadas R. Lim C. T. Dao M. (2018). Febrile Temperature Elevates the Expression of Phosphatidylserine on *Plasmodium Falciparum* (FCR3CSA) Infected Red Blood Cell Surface Leading to Increased Cytoadhesion. Sci. Rep. 8, 15022. doi: 10.1038/s41598-018-33358-2 30302009 PMC6177484

[B217] Zhang J. Reinhart-King C. A. (2020). Targeting Tissue Stiffness in Metastasis: Mechanomedicine Improves Cancer Therapy. Cancer Cell 37 (6), 754–755. doi: 10.1016/j.ccell.2020.05.011 32516585

